# Open ocean and coastal strains of the N_2_-fixing cyanobacterium UCYN-A have distinct transcriptomes

**DOI:** 10.1371/journal.pone.0272674

**Published:** 2023-05-02

**Authors:** María del Carmen Muñoz-Marín, Jonathan D. Magasin, Jonathan P. Zehr

**Affiliations:** 1 Department of Ocean Sciences, University of California Santa Cruz, Santa Cruz, California, United States of America; 2 Departamento de Bioquímica y Biología Molecular, Campus de Excelencia Internacional Agroalimentario, Universidad de Córdoba, Córdoba, Spain; Friedrich Schiller University, GERMANY

## Abstract

Decades of research on marine N_2_ fixation focused on *Trichodesmium*, which are generally free-living cyanobacteria, but in recent years the endosymbiotic cyanobacterium *Candidatus* Atelocyanobacterium thalassa (UCYN-A) has received increasing attention. However, few studies have shed light on the influence of the host versus the habitat on UCYN-A N_2_ fixation and overall metabolism. Here we compared transcriptomes from natural populations of UCYN-A from oligotrophic open-ocean versus nutrient-rich coastal waters, using a microarray that targets the full genomes of UCYN-A1 and UCYN-A2 and known genes for UCYN-A3. We found that UCYN-A2, usually regarded as adapted to coastal environments, was transcriptionally very active in the open ocean and appeared to be less impacted by habitat change than UCYN-A1. Moreover, for genes with 24 h periodic expression we observed strong but inverse correlations among UCYN-A1, A2, and A3 to oxygen and chlorophyll, which suggests distinct host-symbiont relationships. Across habitats and sublineages, genes for N_2_ fixation and energy production had high transcript levels, and, intriguingly, were among the minority of genes that kept the same schedule of diel expression. This might indicate different regulatory mechanisms for genes that are critical to the symbiosis for the exchange of nitrogen for carbon from the host. Our results underscore the importance of N_2_ fixation in UCYN-A symbioses across habitats, with consequences for community interactions and global biogeochemical cycles.

## Introduction

Ocean productivity depends on the availability of nitrogen (N). Dissolved atmospheric N_2_, though plentiful in surface waters, is biologically unavailable to the marine microorganisms that drive ocean productivity [1]. Instead, they largely rely on fixed inorganic N compounds such as ammonium and nitrate, which they rapidly deplete to low levels in the well-lit surface waters of the open ocean gyres. Only some microorganisms can fix N_2_, reducing it to ammonium, and are the main source of N for productivity in terrestrial and aquatic environments [2]. In the marine environment several species of cyanobacteria, including symbionts of diatoms and haptophytes, are known to be important N_2_-fixers. These include the uncultivated unicellular cyanobacteria *Candidatus* Atelocyanobacterium thalassa (UCYN-A) because of their high N_2_ fixation and growth rates [3–5] and potential for N transfer into the food web through grazers [3, 6, 7]. DNA sequencing showed that UCYN-A has a massively streamlined genome [8, 9] that lacks the machinery required for carbon fixation, including photosystem II (PSII) and RuBisCO, as well as other key metabolic pathways, e.g. the entire tricarboxylic acid cycle (TCA) [8, 10, 11]. Later it was discovered that UCYN-A lives in symbiosis with a photosynthetic haptophyte, related to *Braarudosphaera bigelowii*, with which it exchanges fixed N for fixed carbon [10, 11]. Subsequent work showed that disrupting photosynthesis by the host arrested UCYN-A cell division and changed its daily transcription pattern of the key enzyme in N_2_ fixation, the nitrogenase gene (*nifH*) [12]. All these studies show a close relationship between UCYN-A and the host, however the interplay of environmental and host factors on UCYN-A metabolism and distribution is still not known.

Phylogenetic analyses of *nifH*, have shown that there are at least five distinct UCYN-A sublineages [13–16]. The symbioses are globally distributed and include nutrient-enriched coastal waters [4, 17–22], oligotrophic open ocean [3, 16, 23, 24], and low-temperature waters [25–27]. Some UCYN-A sublineages occur more frequently than others on sinking particles [28], which might impact particle-associated communities and the export of fixed N_2_. All of this suggests that UCYN-A diversity and distribution are important for a complete picture of N_2_ fixation in the global ocean.

It has been shown that UCYN-A fixes N_2_ during the day and coordinates N_2_ fixation and general metabolism similarly to other cyanobacteria that fix N_2_ during the day such as *Trichodesmium* and heterocyst-forming cyanobacteria [17]. Critical to this coordination is the protection of nitrogenase from oxygen evolved by photosynthesis by the host, because oxygen irreversibly damages nitrogenase [29, 30]. Although much gene expression in cyanobacteria follows circadian rhythms that are driven by three “clock” (*kai*) genes [31–33], UCYN-A lacks two of the three *kai* genes. Thus, UCYN-A’s mechanism for coordinating expression with that of its host is poorly understood [8]. UCYN-A might be controlled by a novel circadian network or the circadian rhythm of the host, and/or UCYN-A might simply respond to host metabolites [17].

Many essential aspects of the symbioses have not yet been explored, in particular, the metabolism under different nutrient conditions. If UCYN-A depends on metabolite production by the host, does its metabolism differ in nutrient-rich versus poor waters? Metatranscriptomes for UCYN-A1 and UCYN-A2 from nutrient-rich coastal waters were previously analyzed [17]. The present work examines transcriptional patterns in oligotrophic water (characterized by low concentrations of macronutrients and warm surface temperatures). Specifically, we analyzed transcription over diel cycles by UCYN-A sublineages A1, A2, and A3 at Stn. ALOHA, an open-ocean station in the North Pacific Subtropical Gyre (NPSG) and, for UCYN-A1 and A2, compared to diel transcriptional patterns in coastal waters at Scripps Pier.

First, we reason that UCYN-A2 transcripts per cell must have been higher than rates for other UCYN-A sublineages at Stn. ALOHA. Second, we show that highly transcribed genes were similar at both sites. However, when all detected genes were included, striking differences became apparent in the daily expression patterns at Stn. ALOHA and Scripps Pier, as well as among UCYN-A sublineages at Stn. ALOHA. Fourth, when we looked at genes with significant 24 h periodic expression ("diel genes"), we observed differences in the number and timing across habitats, with some intriguing exceptions for genes involved in nitrogen fixation. Our findings offer clues to UCYN-A1/2/3 habitat preferences and regulation by their respective hosts in open-ocean versus coastal environments, just when culture studies for some UCYN-A sublineages have become possible.

## Materials and methods

### *In situ* sampling

#### UCYN-A at Stn. ALOHA (diel samples)

Samples were collected from CTD casts at Stn. ALOHA (22° 45’ N, 158° 0’ W at 45 m) during the C-MORE Cruise C-20 (http://hahana.soest.hawaii.edu/hot/cruises.html) between 6th and 10th April, 2015. No permits were required to collect samples at Stn. ALOHA, which is in international waters. Seawater was collected in 12-L polyvinylchloride bottles affixed to a rosette sampler equipped with Sea-Bird 911+ conductivity, temperature, and pressure sensors.

A total of 16 samples were collected to cover a full light-dark cycle for the diel expression analysis (below) with 8 time points and 3 h intervals. Time points were as follows: 9:00-L3, 12:00-L6, 15:00-L9, 18:00-L12, 21:00-D3, 00:00-D6, 03:00-D9, 06:00-2D12, 09:00-2L3 and 12:00-2L6, where L and D indicate the light and dark period, respectively, 2L and 2D the second light-dark cycle, and the number the corresponding hours into the light or dark period. Two replicates were collected for each time point except for D3, D6, as well as L3 and 2L3. The first and second experiments were considered biological replicates. S1 Table shows the collection dates and the total daylight for samples collected at Stn. ALOHA and Scripps Pier (S1 Table). Environmental conditions for Stn. ALOHA and Scripps Pier during the experiment are shown in S2 Table.

Samples were collected by filtering a total of 500 mL from each seawater replicate through 0.22 μm pore-size, 47 mm diameter Supor filters (Pall Corporation, Port Washington, NY, USA) using a peristaltic pump. Filters were placed in sterile 2 mL bead-beating tubes with sterile glass beads, flash-frozen in liquid nitrogen and stored at -80°C until extraction.

#### UCYN-A at Scripps Pier (diel samples)

Surface seawater samples were collected using a bucket from the end of the Ellen Browning Scripps Memorial Pier in La Jolla, CA, USA on July 29 and 30, 2014 [17]. A total of 16 samples covered a full light-dark cycle with 8 time points at 3 h intervals (S1 Table).

#### UCYN-A in the South Atlantic (Tara Oceans non-diel samples)

The Tara Oceans metatranscriptomes used in our analysis were from surface seawater samples collected at South Atlantic stations 76 and 78. The samples were filtered into four size fractions (0.2–3, 0.8–5, 5–20 and >0.8-μm pore-size filters) [34]. Filters were kept at −20 °C for ∼4 weeks on the schooner and then at −80 °C in the laboratory until nucleic acid extraction [34]. DNA and cDNA libraries for these samples underwent paired-end Illumina HiSeq2000 sequencing [34]. Reads from UCYN-A1 and UCYN-A2 were identified by fragment recruitment to reference genomes [34]. For our analysis we used transcript counts that were normalized by total metagenomic read counts for each UCYN-A sublineage, published in Supplementary Data 1 and 2 of [34].

### RNA extraction and processing for hybridization to the microarray

Environmental RNA containing transcripts from UCYN-A cells was extracted using the Ambion RiboPure Bacteria Kit (Ambion^®^, ThermoFisher) and treated using the Turbo-DNA-free^TM^ DNase Kit (Ambion^®^, ThermoFisher) following the procedure described in [17].

RNA purity, concentration and quality were determined using a NanoDrop 1000 (Thermo Scientific, Waltham, MA, USA) and a 2100 Bioanalyzer (Agilent Technologies, Santa Clara, CA, USA) using the RNA 6000 Nano kit (Agilent Technologies). Only samples with RNA Integrity Number >7.0 and ratios of A260/A230 and A260/A280 ≥1.8 were processed further.

Double-stranded (ds) cDNA was synthesized and amplified following the procedure described in Muñoz-Marín et al., 2019. Briefly, 400 ng of RNA from each sample was used. Spike-in transcripts were added to each sample to monitor amplification performance, specifically, 1μL of a 1:100 dilution of External RNA Control Consortium (ERCC) RNA spike-in mix 1 (Ambion) [35] (S1 Fig). The dilution corresponded to 4.7 aM of spike-in ERCC-0016. The amplified cDNA was purified with the GenElute PCR cleanup kit (Sigma-Aldrich), and the quality and quantity of ds-cDNA was determined with NanoDrop 1000 and a 2100 Bioanalyzer using the Agilent DNA 7500 kit (Agilent Technologies). Four hundred ng of total RNA yielded on average 12 μg of ds-cDNA. The labeling and hybridization of cDNA samples (1.0 μg of ds-cDNA) to the microarray was done at the Roy J. Carver Center for Genomics (CCG) Facility (University of Iowa, Iowa city, Iowa, USA) according to the Agilent Technology protocol for arrays.

### Design of the UCYN-A array

The UCYN-A oligonucleotide expression array targeted nearly all known genes from UCYN-A1 (1195 of 1199 genes) and UCYN-A2 (1244 of 1246 genes) with oligonucleotide probe sequences ("probes"). These UCYN-A1 and UCYN-A2 probes were designed using the eArray Web-based tool (Agilent Technology Inc.; https://earray.chem.agilent.com/earray/) with the gene sequences obtained from the National Center of Biotechnology Information (NCBI; https://www.ncbi.nlm.nih.gov) using accessions NC_013771.1 for UCYN-A1 and JPSP00000000.1 for UCYN-A2. Usually, 4 to 6 probes of 60 nucleotides (nt) in length were designed for most genes, and a total of 6,088 probes (1,195 genes) and 6,324 probes (1,244 genes) were designed for UCYN-A1 and UCYN-A2, respectively. In this work, we used the UCYN-A probes designed previously by Muñoz-Marin et al., (2019).

For UCYN-A3 314 genes were targeted, all of them reconstructed in [16]. A total of 1351 probes, 60 nt in length, were designed for UCYN-A3 with the eArray web-based tool using a similar approach to that described above. Usually, 4 to 6 distinct probes represented each of the 314 genes.

#### In silico check for cross hybridization

UCYN-A1 and A2 genomes share orthologs for 97% of protein coding genes in UCYN-A1. These orthologs are on average 86% amino identical, and mainly 80–90% nucleotide identical (nid) [36]. One exception is the N_2_ fixation gene *nifH* (95% nid) [36]. Reconstructed UCYN-A3 genes were <95% nid to the UCYN-A1 and A2 reference genomes, but generally recruited reads were >80% nid [16]. Therefore, the UCYN-A1, A2, and A3 probes were tested *in silico* for possible cross-hybridization. First, we checked for cross-hybridization to non-UCYN-A microbes. The probe sequences were used as BLASTN queries against the following available nucleotide databases in June 2014: Community Cyberinfrastructure for Advanced Microbial Ecology Research and Analysis (CAMERA [37], now available in iMicrobe [38]), Marine microbes, Microbial Eukaryote Transcription and Non-redundant Nucleotides. Candidate probes were rejected if they had BLASTN alignments that were ≥ 95% nid over the full probe length to non-UCYN-A sequences. These alignment criteria simulated the hybridization specificity of Agilent SurePrint technology which was used for the microarrays and allows up to a 5% nt mismatch over the whole probe.

Next, we checked that probes would only hybridize to the intended UCYN-A sublineage. Probes were clustered using CD-HIT-EST [39] at 95% nid. A probe was rejected if it was in a cluster with multiple UCYN-A sublineages or genes. A few exceptions were made for probes from highly conserved genes (such as the nitrogenase gene, *nifH*).

The surprising result in section 1 prompted an additional check for probe specificity. We simulated hybridization *in silico* by aligning UCYN-A1, A2, and A3 reference sequences to the microarray probe sequences using blastn-short in the BLAST+ tool suite (v2.6.0). RefSeq assemblies GCF_000025125.1 and GCF_000737945.1 were used for UCYN-A1 and A2, respectively. For UCYN-A3, metagenomic contigs were kindly provided by Francisco Cornejo-Castillo (unpublished). Alignments to the unintended sublineage at ≥95% nid and over ≥95% of the target probe were considered cross-hybridizations, following Agilent SurePrint specificity [40]. Such cross-hybridizations were rare and are summarized in S2 Fig. In particular, for UCYN-A2 a total of 210 probes (3.3% of 6324 total UCYN-A2 probes on the array) cross-hybridized, all at <100% nid and mainly (205 probes) to UCYN-A3. It is implausible that so few cross-hybridizing probes would have impacted detection of UCYN-A2 genes: Gene intensities (in each sample) were based on averages over the gene’s probe set, and the averages would have been dominated by the majority of UCYN-A2 probes which did not cross-hybridize.

In summary, 6088 probes for 1195 UCYN-A1 genes, 6324 probes for 1244 UCYN-A2 genes, and 1351 probes for 314 UCYN-A3 genes were chosen (S3 and S4 Figs). Each of these 13,763 total experimental probes were replicated 4 times at different microarray coordinates (55,052 experimental features). A total of 96 ERCC spike-in control probes were at 7025 random feature locations on the microarray. There were also 899 standard control features defined in the Agilent control grid (IS-62976-8-V2_60Kby8_GX_EQC_201000210). For every microarray design format and species, Agilent includes a default species-specific control grid. The grids contain positive control probes that were designed against the endogenous sequence for the species, and are used for image orientation and to assess whether the sample was labeled. The grids also include negative control probes (e.g., structural hairpins which do not hybridize to anything) which are used to measure the background [41]. The final design of the microarray included ca. 62,976 features and was synthesized as 8 arrays per slide on two slides for our 16 total samples.

The probe sequences are available at NCBI Gene Expression Omnibus (GEO) under accession GSE206403.

### Microarray analyses

#### Normalization and gene detection

Microarray data were processed with R (www.R-project.org) v4.0.4 and the MicroTOOLs R package (https://www.jzehrlab.com/microtools), which has been used previously to analyze marine microbial community metatranscriptomes targeted by the MicroTOOLs array design [42, 43]. In the present study we substituted in the UCYN-A microarray design (NCBI GEO accession GPL32341). All 16 microarrays passed quality checks from the arrayQualityMetrics R package v3.44 [44]. Probe intensities were normalized across samples by quantiles [45] and then converted to gene intensities ("transcript levels" in Results, log_2_ scale) using robust multi-array averaging [46] using the affyPLM R package v1.64 [47]. Then genes were detected within each sample if they had a signal-to-noise ratio (SNR, or *z*-score) that was >5, with noise defined as the median intensity of the bottom 10% of genes in the sample. A total of 2753 genes were detected based on SNR>5. However, we also required detected genes to be higher than the least concentrated spike-in control (ERCC-00048) in >3 samples (S1 Fig). This additional requirement was satisfied based on either of two comparisons: (i) array intensities of the gene versus the ERCC; (ii) predicted transcript counts of the gene versus the ERCC, based on a linear model for transcript counts dependent on array intensity, created with the R function lm. After filtering based on ERCC spike-ins, the total number of detected genes was 1939 (S3 Table).

For the Stn. ALOHA as well as the Scripps Pier microarray designs, 91% of genes were represented by ≥3 probes that covered different parts of the gene. Genes with fewer probes are not likely to have impacted the analyses (S1 File).

Raw and normalized microarray data for UCYN-A at Stn. ALOHA were submitted to NCBI GEO under accession GSE206403. The script for processing the raw microarray data from Stn. ALOHA into detected genes, runMicroTOOLsPipeline.R, is available in a GitHub repository for this study (https://github.com/jdmagasin/UCYN-A-metatranscriptomes). The repository also includes an R image, workspace.Rdata, which contains all the metatranscriptomes used in this work. Microarray data for UCYN-A at the Scripps Pier was deposited at GEO under accession GSE100124.

#### Diel genes

To identify genes with significantly 24 hour periodic transcript levels ("diel genes"; false discovery rate <0.25), we used the fdrfourier function in the R package cycle v1.42 with AR1 background models [48] and 1000 background data sets. The Fourier scores, false discovery rates, and diel classifications are in S3 Table columns Fourier_score, Fourier_FDR, and Diel, respectively. After standardizing transcript levels (0 mean, unit variance), diel genes were clustered with the pvclust R package v2.2 (correlation based distances, clustering by centroids, 1000 bootstraps, cluster significance alpha = 0.95; [49]). Assigned clusters are indicated in column Diel_cluster of S3 Table, with most diel genes in either a cluster with sunrise peaks (D12, cluster "sunrise_peak_edge.172") or sunset peaks (L12, cluster "sunset_peak_edge.174"). Code for identifying diel genes is within findCyclicGenes.R in the GitHub repository for this study.

### Environmental data and calculations

CTD data for the Stn. ALOHA metatranscriptomes were downloaded from the C-MORE CTD Extraction web site (https://hahana.soest.hawaii.edu/cmoreDS/cextraction.html) by selecting the HOE Legacy 1 Cruise (KM1503). Times in the cruise event log (https://hahana.soest.hawaii.edu/hoelegacy/documents/documents.html) for casts 11–20 matched the time points for our samples. Therefore, CTD data from casts 11–20 at 45 dbar were used for analyzing the metatranscriptomes (S2 Table).

Scripps Pier samples were not collected during a cruise so there was no CTD data to match each time point. Surface temperature and salinity data were obtained from the UC San Diego Shore Stations Program Data Archive (https://library.ucsd.edu/dc/collection/bb4719748r) specifically the data for Scripps Pier (https://library.ucsd.edu/dc/object/bb4003017c) ([50]; S2 Table). These data had only one measurement per day and were not used for analysis. However, we included the July 2014 monthly averages from Scripps Pier in sheet two of S2 Table along with CTD averages from Stn. ALOHA for comparison.

The National Oceanic and Atmospheric Administration (NOAA) Solar Calculator (https://gml.noaa.gov/grad/solcalc/) was used to calculate daylight hours and times of sunrise and sunset at Stn. ALOHA and Scripps Pier on the days of sample collection (S2 Table).

For Stn. ALOHA, CTD data from all casts (1–20) were used to calculate the mixed-layer depths based on a potential density (σ_θ_) offset of 0.03 kg/m^3^ relative to 10 dbar [51]. The mean and standard deviation were used for analysis (S2 Table and S5 Fig).

CTD data were fitted to the Stn. ALOHA ordination from non-metric multidimensional scaling (NMDS, described below).

### Statistical analyses

#### Section 1: Comparison of UCYN-A2 and UCYN-A1 transcript levels

Previously published Illumina metatranscriptomic data from South Atlantic Tara Oceans Stn. 76 and 78 (described above; [34] were used to corroborate some results obtained with the microarrays. Gene loci were used to map genes in the Tara study to Gene Identifiers (GIDs) in the UCYN-A microarray design, which allowed transferring pathway and other annotations from the microarray to the Tara data. One-tailed *t*-tests were used to see if UCYN-A2 transcript counts were greater than UCYN-A1 counts in the 0.8 μm size fraction from Tara Stn. 78, the only sample in which UCYN-A1 and UCYN-A2 were both abundant [34]. The *t*-tests were either paired with 272 genes detected in the >0.8 μm size fraction, or unpaired with 1529 genes from UCYN-A2 and 2008 genes from UCYN-A1 detected in any size fraction (0.2–3, >0.8, or 5–20 μm). Code for both tests are available in statistical_tests.R in our GitHub repository.

#### Section 2: Highly transcribed genes

**Fig 1:**
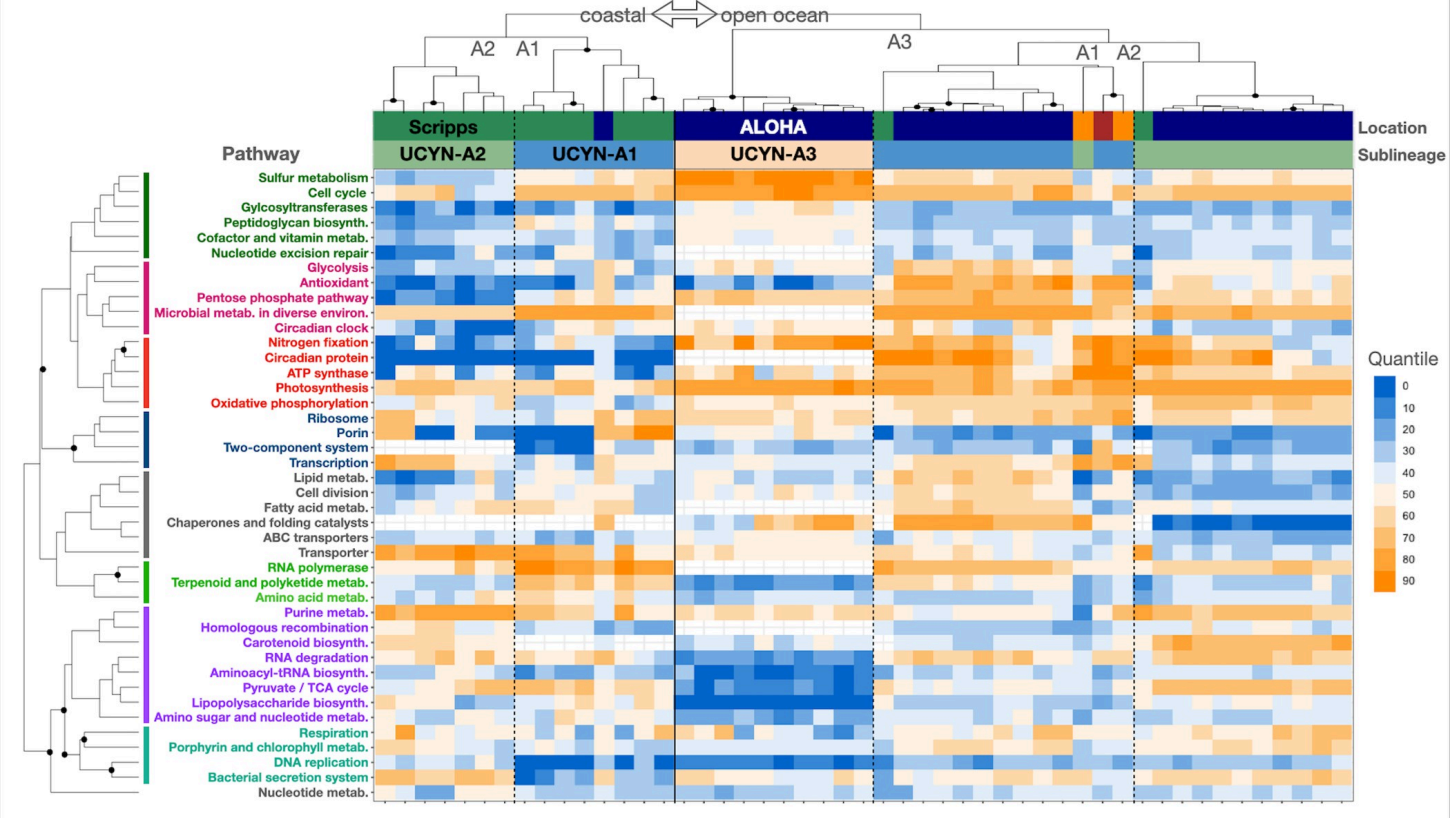
The heat map shows that expression is driven first by location (Stn. ALOHA, Scripps Pier, Tara SAtl), then by sublineage. Each column is a specific sublineage at a location and is standardized (quantiles). Cell colors are the quantile for the median intensity of the genes in the pathway. Sample and pathway clusters with solid discs were strongly supported (Methods). In the Location bar, samples from South Atlantic Tara Oceans stations 76 (brown) and 78 (orange) are shown.

*(i) Combining microarray and Tara metatranscriptomes for Fig 1*. Fig 1 includes microarray metatranscriptomes from Stn. ALOHA and Scripps Pier as well as sequenced metatranscriptomes from Tara Stns. 76 and 78. It is the only case where the Tara and microarray data were processed together. To combine the data sets, transcript levels (intensity or read count) were converted to quantiles for detected pathways. First, for each sample and strain, pathways levels were calculated as the median transcript level (intensity or read count) of the detected genes in the pathway. Pathway levels, for each strain and in each sample, were then converted to quantiles at 5% intervals. For legibility, Fig 1 includes only the pathways that were detected in more than one third of samples. The retained pathways were hierarchically clustered using pvclust as described above except that average linkage clustering was used. Samples were hierarchically clustered the same way but standardizing by quantiles was unnecessary.

**Fig 2:**
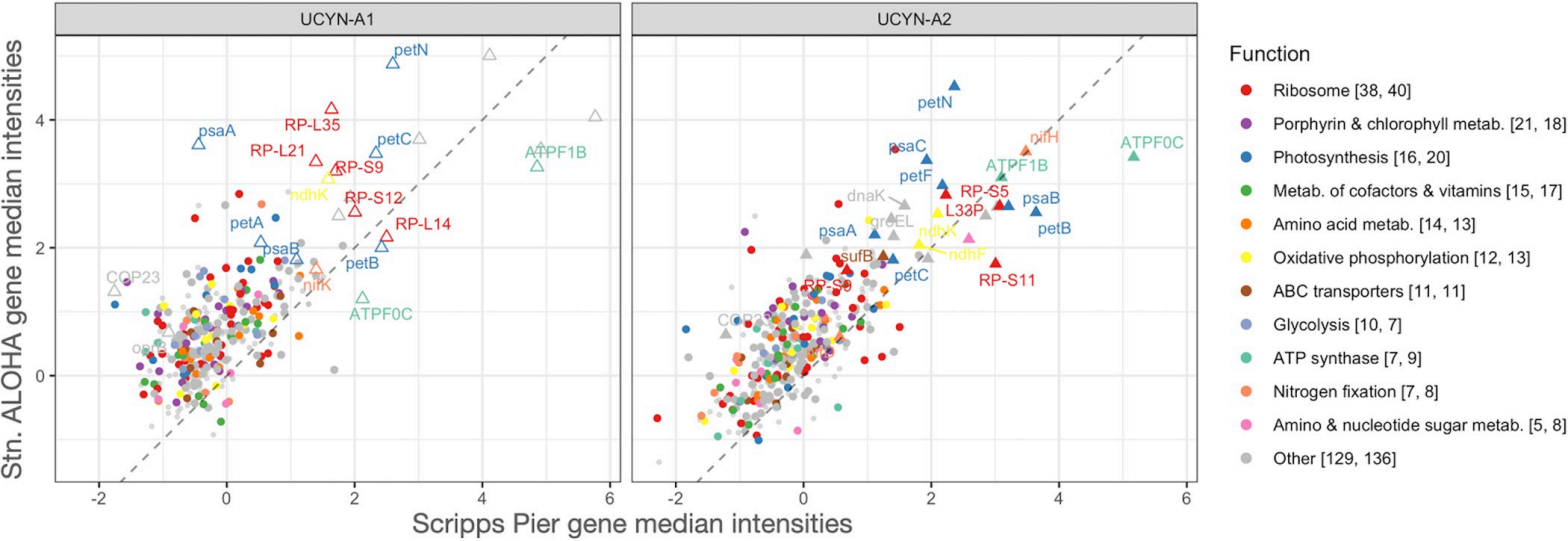
Shown are all 760 genes (*n*_*UCYN-A1*_ = 368, *n*_*UCYN-A2*_ = 392) that were detected at both Stn. ALOHA and Scripps Pier. Axes are the standardized transcript levels (Methods) and the dashed reference lines indicate equal standardized transcript levels at both sites. Genes are above the reference lines which is consistent with the hypothesis that the coastal study favored the detection of highly transcribed UCYN-A genes but missed less transcribed genes (main text). Genes are colored by pathway and the legend includes in brackets the number of cross-site detected genes for UCYN-A1 and A2. Genes that had transcript levels in the top 10% at both sites for UCYN-A1 are named and shown as open triangles. Top genes for UCYN-A2 are shown with filled triangles, and names shared with UCYN-A1 indicate orthologs.

*(ii) Representing cross-site detected in Fig 2*. Data sets from Stn. ALOHA and Scripps Pier were normalized separately. To enable highly transcribed genes to be compared across sites as in Fig 2, we therefore had to standardize transcript levels. For each detected gene at each site, the standardized transcript level was the median of the gene’s *z*-scores from all samples at the site.

*(iii) Higher percentages of detected genes at Stn*. *ALOHA than at the coastal site*. To help understand why more genes were detected at Stn. ALOHA than at Scripps Pier for both UCYN-A1 and UCYN-A2, we checked if detection at Scripps Pier might have favored highly transcribed genes. To do this we used separate Welch two-sample *t*-tests for each strain to see if genes detected at both sites had significantly higher median transcript levels than genes detected at Stn. ALOHA but not at Scripps Pier. Code for these tests is available in statistical_tests.R in our GitHub repository.

*(iv) NMDS and subsequent fitting of environmental data*. To connect pathway transcript levels to environmental data, two-dimensional non-metric multidimensional scaling (NMDS) was done for Stn. ALOHA metatranscriptomes using the metaMDS function in the R package vegan (v2.5–7; [52]). NMDS was similarly done for Scripps Pier to enable comparison to Stn. ALOHA. For both NMDS analyses we included 760 genes that were detected at both sites. The Stn. ALOHA NMDS also included 107 diel genes that were detected only at Stn. ALOHA. For both NMDS analyses we used Euclidean distances between gene transcript levels. For Stn. ALOHA, CTD data were fit to the NMDS ordination using the envfit function in vegan. The approach produced models that maximized the correlations between the Stn. ALOHA NMDS ordination and the dependent environmental variables. Additionally, envfit performed significance tests for each fitted environmental variable (by comparison to correlations for 999 permutations of the environmental variables). We considered an environmental variable significant if the *p*-value for its squared correlation coefficients was <0.05. No CTD or other time-point matched environmental data was available for the Scripps Pier samples.

#### Section 3: Times of peak transcript levels

**Fig 3:**
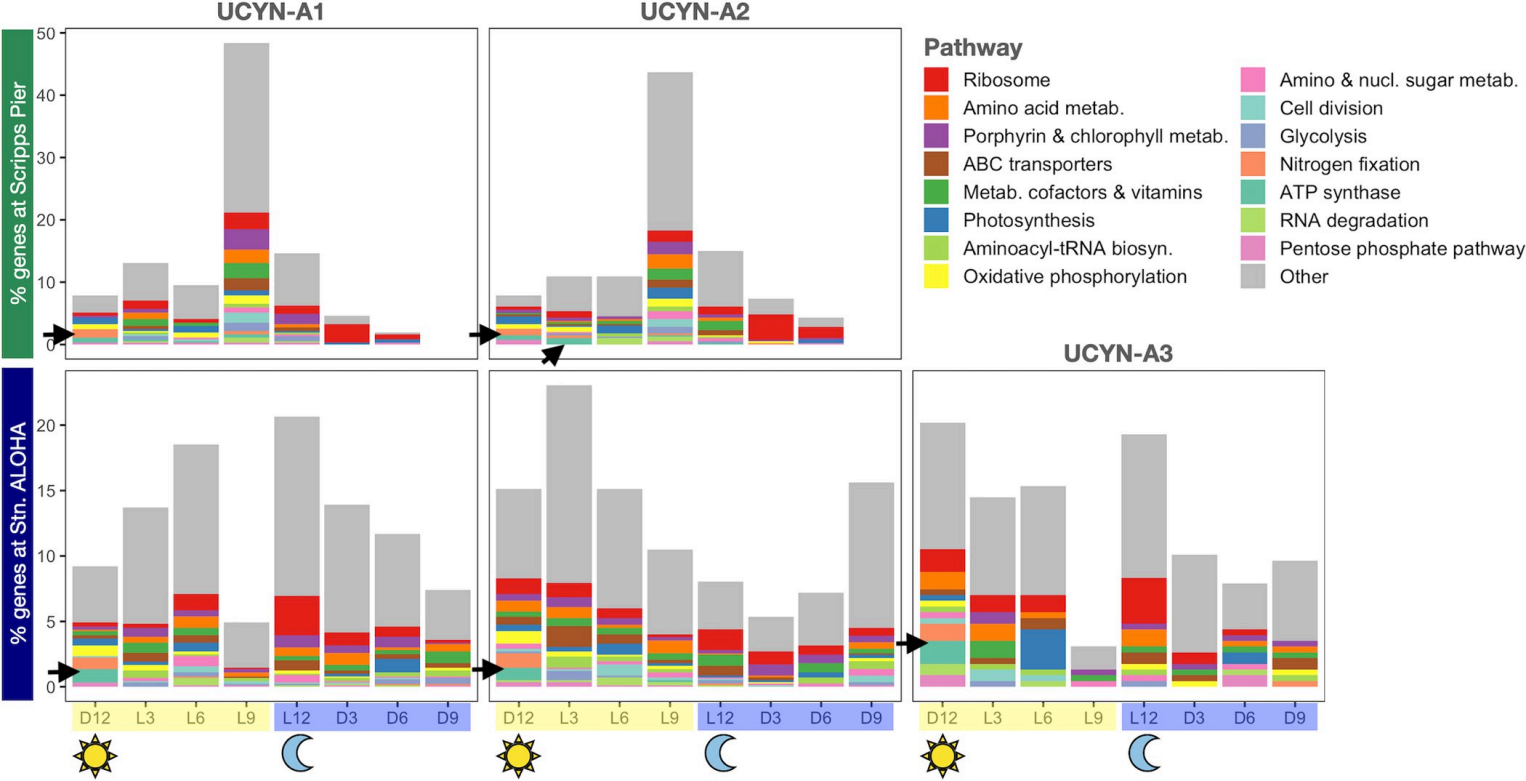
All detected genes at Stn. ALOHA and Scripps Pier are shown at the time of day when their transcript levels peaked. For each habitat and sublineage, the histogram shows the time of day when each detected gene had its highest average transcript level (Methods). The *y* axes show the percentage of detected genes from the sublineage. Black arrows indicate genes important to nitrogen fixation (*nif* genes and ATP synthases) which always peaked near sunrise (D12).

This section used no statistical tests. Histograms were created for the times of highest transcript levels (Fig 3) and lowest transcript levels (S6 Fig) for all detected genes at Stn. ALOHA and Scripps Pier. Within each site, for each gene the transcript levels were averaged (mean) over replicates, and also from days 1 and 2 (e.g. L6 and 2L6) prior to identifying the peak and trough times.

#### Section 4: Diel genes at Stn. ALOHA and Scripps Pier

To test whether diel genes were contingent on habitat, we used Fisher’s exact test [52] as implemented by the R function fisher.test, with alternative = "two.sided" and restricted to the 760 cross-site detected genes: 679 non-diel and 81 diel at ALOHA; and 111 non-diel and 649 diel at Scripps Pier. The null hypothesis that diel genes were not contingent on habitat was rejected (*p<0*.*001*). Code for this test is in statisticalTests.R.

Additionally, we checked whether differences in the number of diel genes at Stn. ALOHA and Scripps Pier were impacted by the different numbers of time points used for each study (10 and 8, respectively), by repeating the Fourier score analysis using the 8 shared time points and only the genes detected at both sites (S1 File).

For Stn. ALOHA, the R function cor was used to calculate Pearson correlations between transcript levels in each sample and corresponding CTD data for potential density, salinity, temperature, photosynthetically active radiation (PAR), oxygen concentration, and chlorophyll concentration (CTD data in S2 Table, correlations shown in S7 Fig).

## Results and discussion

### 1. UCYN-A2 had higher transcripts per cell than other sublineages at Stn. ALOHA

The UCYN-A population at Stn. ALOHA was comprised mainly of the open ocean sublineages UCYN-A1 (87% of amplified *nifH* gene sequences) and UCYN-A3 (9.4%), and very little of the coastal sublineage UCYN-A2 (~0.7%) based on a previous study that used the same samples [16]. It was therefore surprising that UCYN-A2 had microarray probe intensities as high as UCYN-A1 and A3 (S8 Fig) and was as robustly detected (75%, 66%, and 73% of gene targets detected for UCYN-A1, A2, and A3, respectively; S3 Table). These results are not likely explained by cross-hybridization because *in silico* simulations indicated that only ~3% of the UCYN-A2 probes could hybridize to transcripts from UCYN-A1 or UCYN-A3 using Agilent SurePrint technology (only transcripts >95%id over the whole probe length will hybridize). However, it is possible that the array detected a sublineage with mainly >95% nucleic acid identity to the Scripps Pier UCYN-A2 isolate (SIO64986) that was used to design the probes. Genetic differences between SIO64986 and the sublineage at Stn. ALOHA might explain the slightly higher frequency of low-intensity probes for UCYN-A2 compared to UCYN-A1 and A3 (S8 Fig) and slightly lower percentage of gene targets detected.

The high transcript levels for UCYN-A2 despite its rarity suggest that UCYN-A2 produced more transcripts per cell than other sublineages at Stn. ALOHA. This was corroborated by comparing UCYN-A2 and UCYN-A1 transcript counts from South Atlantic (Tara Oceans Stn. 78) surface water metatranscriptomes. The authors showed that 1.6× more *nifH* transcript sequences came from UCYN-A2 than UCYN-A1 (in the >0.8 μm size fraction; [34]. Additionally, we found that UCYN-A2 (at Stn. 78 in the >0.8 μm size fraction) had on average 3.8× more transcript sequences than UCYN-A1 for genes detected for both sublineages (*p<0*.*001*, one-tailed paired *t*-test, *n* = 272 gene observations). The UCYN-A2 counts were also higher for genes detected for either sublineage in any size fraction (0.2–3, >0.8, or 5–20 μm; *p<0*.*001*, one-tailed unpaired *t*-test, *n*_*A2*_ = 1529 gene observations, *n*_*A1*_ = 2008 gene observations). Thus, in the oligotrophic NPSG and south Atlantic, UCYN-A2 cells were transcriptionally more active than UCYN-A1, which might imply higher N_2_ fixation rates.

Indeed, higher rates of N_2_ fixation by UCYN-A2 compared to UCYN-A1 have been measured in the oligotrophic tropical North Atlantic [4]; the Arctic [25]; and even in nutrient-rich southern California coastal waters [4, 53], where the highest UCYN-A2 N_2_ fixation rates occurred after upwelling had increased nutrient concentrations (nitrate+nitrite and phosphate) and decreased temperature [4]. This suggests that higher fixation by UCYN-A2 compared to UCYN-A1 occurs across habitats and is not likely a response by the UCYN-A2 symbiosis to low nutrients. Instead, the UCYN-A2 host might require more fixed N_2_ than the UCYN-A1 host [34]. Consistent with this hypothesis, the UCYN-A2 host, which is closely related to the UCYN-A1 host based on 18S rRNA gene sequences, is larger (length ~3–4 μm versus ~2 μm for the UCYN-A1 host) [13] and contains more symbionts than the UCYN-A1 host [16, 34].

### 2. Highly transcribed genes were similar across sublineages and environments

At Stn. ALOHA, UCYN-A sublineages had highest transcript levels for the same genes and pathways including photosynthesis (*psaAB* as well as cytochrome b_6_f complex genes *petBC*), ATP synthases, N_2_ fixation (*nifDK*), ABC transporters, cell cycle (*clpP*) and division (*ftsH*), glycolysis (FBA), respiration (*coxB*), and ribosomal proteins (Figs 1 and 2 and S3 Table). UCYN-A1 and A2 shared several other top genes for the metabolism of porphyrin and chlorophyll (*hemL* and an unnamed enzyme [E1.14.13.81]), oxidative phosphorylation (*ndhCK*), bacterial secretion system (*secY*), carbohydrate porins (*oprB*), RNA degradation (*dnaK*), and the circadian oscillating protein 23 (COP23). The microarray lacks these genes for UCYN-A3.

The most highly transcribed genes at Stn. ALOHA and Scripps Pier were similar for UCYN-A1, as well as for UCYN-A2, and came from several of the pathways already mentioned: photosynthesis, cytochrome b_6_f complex, ATP synthases, N_2_ fixation, and oxidative phosphorylation (Fig 2). Moreover, we identified genes from these pathways among the top transcribed by both UCYN-A1 and A2 in the South Atlantic Ocean [34]. The high transcript levels for genes in these pathways across sublineages and environments suggests that they are fundamental to UCYN-A symbioses, perhaps because they function in the regeneration of reductant and ATP needed for N_2_ fixation as proposed by Muñoz-Marín et al. 2019.

We also compared all detected genes at Stn. ALOHA and Scripps Pier. Far more genes were detected in the present work (1939 genes) than at Scripps Pier (762 genes; S3 Table). This is likely explained by higher UCYN-A transcript relative abundances in the oligotrophic community compared to the coastal [54]. If so, then the coastal study favored the detection of highly transcribed UCYN-A genes but missed less transcribed genes, which were detected only at Stn. ALOHA. Consistent with this hypothesis nearly every gene detected at Scripps Pier was also detected at Stn. ALOHA (760 of 762 genes), and the 760 cross-site detected genes (*n*_*A1*_ = 362 and *n*_*A2*_ = 398) had significantly higher median transcript levels than the other 1179 genes detected only at Stn. ALOHA (*p<0*.*001* in Welch’s *t*-tests performed separately for UCYN-A1 and A2; Fig 2). A similar result was seen for UCYN-A3: Transcript levels from UCYN-A3 genes that had orthologs to cross-site detected genes were significantly higher (*p<0*.*001* in Welch’s *t*-test comparison of levels from 103 UCYN-A3 genes with cross-site orthologs to 44 without). Altogether these observations indicate that (1) more of each UCYN-A metatranscriptome was detected at Stn. ALOHA than at Scripps Pier, and (2) highly transcribed genes and pathways were similar across UCYN-A sublineages and environments.

Correlation analyses also showed overall similar transcription intensities across sites but additionally suggested that the UCYN-A1 symbiosis was more sensitive to habitat (Figs 2 and S9). Each sublineage (considered separately) tended to transcribe genes in similar order of intensity across sites (Spearman’s ρ_*A1*_ = 0.57, ρ_*A2*_ = 0.70, and *p<0*.*001* for both; Fig 2). Moreover, pathways were often highly correlated across sites (S9 Fig). For example, transcript levels for ribosomal genes were highly correlated across sites for UCYN-A1 (Pearson’s ρ_A1_ = 0.72) and also for UCYN-A2 (ρ_A2_ = 0.68), as were photosynthesis genes (ρ_A1_ = 0.68, ρ_A2_ = 0.78), and ATP synthases (ρ_A1_ = 0.91, ρ_A2_ = 0.91). Interestingly, the pathway correlations for UCYN-A1 were mainly lower than for UCYN-A2, consistent with the overall Spearman correlations for gene ranks, which might indicate that UCYN-A1 or its host was more impacted by habitat changes than was UCYN-A2 or its host. For example, UCYN-A1 had lower cross-site correlations for N_2_ fixation (ρ_A1_ = 0.54, ρ_A2_ = 0.97), the pentose phosphate pathway (ρ_A1_ = 0.41, ρ_A2_ = 0.64) and RNA degradation genes (ρ_A1_ = 0.11, ρ_A2_ = 0.91). For RNA degradation genes the striking difference was due to *groEL* (GID.646530537 and GID.2528847911 in UCYN-A1 and A2, respectively), which were highly transcribed across sites for UCYN-A2 but only at Scripps Pier for UCYN-A1. Most cyanobacteria (including UCYN-A1 and A2) have two *groEL* genes [55, 56], which encode chaperonins within which proteins can refold, alleviating the aggregation of denatured proteins under conditions of heat and other stresses [57]. In a culture study of a cyanobacterial diazotroph, overexpression of *groEL* was shown to support increased N_2_ fixation during salt stress [58]. Possibly the lower *groEL* transcript levels for UCYN-A1 compared to A2 at Stn. ALOHA are due to different or faster responses to light or temperature at 45 m. Porphyrin and chlorophyll metabolism genes were weakly correlated across sites for both sublineages (ρ_A1_ = 0.30, ρ_A2_ = 0.20). If cross-habitat transcriptomic data were available for the hosts, we could compare host and symbiont pathways that responded to habitat change. This would provide clues as to whether the UCYN-A responses we observed were due to habitat or were host-mediated.

### 3. Transcripts peaked at different times for Stn. ALOHA sublineages, except for genes involved in N_2_ fixation

We looked at the times of day of highest ("peak") and lowest transcript levels for all detected genes. At Stn. ALOHA peak times differed by sublineage and were mainly at sunset for UCYN-A1 (21% at L12), near sunrise for UCYN-A2 (54% from D9 to L3), and at sunrise or sunset for UCYN-A3 (20% at D12, 19% at L12; Figs 3 and S10). Curiously, all three UCYN-A sublineages had increasing percentages of genes peaking from night through sunrise, but with a 3 h lag between sublineages: Increases started at D3 for UCYN-A2, at D6 for UCYN-A3, and at D9 for UCYN-A1 (S10 Fig). In contrast, at Scripps Pier genes tended to peak for both UCYN-A1 and A2 in the late afternoon (L9, 48% for UCYN-A1, 44% for UCYN-A2; Fig 3 and S10 Fig). Looking at the 760 cross-site detected genes, peak times changed from Scripps Pier to Stn. ALOHA for most genes from UCYN-A1 (86% of genes) and UCYN-A2 (82%), often by 6 h or more (47% and 51%, respectively). Photosystem I gene *psaA* peaked at midnight (D6) at Scripps Pier but at midday (L6) at Stn. ALOHA for both sublineages. Moreover, the times of lowest transcript levels varied by sublineage at Stn. ALOHA but were mainly at midnight at Scripps Pier (D6, 83% for UCYN-A1, 73% for UCYN-A2; S6 Fig). Altogether, these results suggest that conditions of lower light (45 m, 14–19% of surface PAR; [59]) and nutrients at Stn. ALOHA, compared to Scripps Pier, drove UCYN-A sublineages to have different times for highest as well as lowest transcriptional activity for most genes.

The exceptions were 122 cross-site genes from UCYN-A1 and A2 (*n*_*A1*_ = 52, *n*_*A2*_ = 70) that had peak transcript levels at the same time of day at Stn. ALOHA and Scripps Pier (S4 Table). For both sublineages many of these genes had roles in N_2_ fixation or photosynthesis, including cytochrome b_6_f complex, ferredoxins, ATP synthases, oxidative phosphorylation, and the metabolism of porphyrin and chlorophyll. These same pathways were identified above as highly transcribed at both sites (Fig 2). NMDS analyses also suggested that pathways associated with N_2_ fixation (*nif* genes, COP23, cytochrome b_6_f complex, ATP synthases, and oxidative phosphorylation genes) were highly transcribed in the morning at both sites (S11 Fig) [17]. Therefore, the maintained peak times across sites further support that N_2_ fixation is fundamental across UCYN-A symbioses and environments.

Lower light and nutrients might have contributed to the timing differences, but the exceptions just described suggest that host interactions were important. First, UCYN-A genomes share mostly the same genes among their highly streamlined genomes [8]. Moreover, the vast majority of protein coding genes shared by UCYN-A1 and A2 are thought to have evolved under purifying selection within the respective hosts, with niche adaptation (positive selection) attributed to none of the shared genes [34]. Second, genes critical to the symbiosis (the exchange of N for C) were the exceptions which maintained the same schedule across sites and UCYN-A sublineages (N_2_ fixation genes as well as ATP synthase genes), which parallels morning peak times for carbon fixation / metabolism genes from haptophytes in the NPSG [60, 61] and California coast [54]. Third, at Stn. ALOHA Gradoville et al. (2021) concluded that light did not impact UCYN-A1 nitrogenase transcription because *nifH* transcript levels did not change from the surface to 100 m (though N_2_ fixation rates decreased), and they observed morning peaks for UCYN-A1 *nifH* transcript levels across depths [62] as in previous studies [13, 63]. Thus, it seems more plausible that the hosts could drive the striking differences in timing for most genes yet maintain morning peaks for genes that underpin the exchange of C and N across different nutrient and light conditions. Host-mediated or not, our results provide evidence for a different regulatory mechanism for genes thought to be essential to UCYN-A symbioses.

### 4. At Stn. ALOHA UCYN-A had fewer diel genes and transcripts peaked more often at sunrise or sunset compared to Scripps Pier

Temporal gene expression profiles can provide insights into gene function and regulation. To identify genes with 24 h periodic expression ("diel genes"), we used a method which calculates Fourier scores for each detected gene and assesses significance (false discovery rate < 0.25) by comparison to scores from a background model [48], similar to the previous coastal study [17].

**Fig 4:**
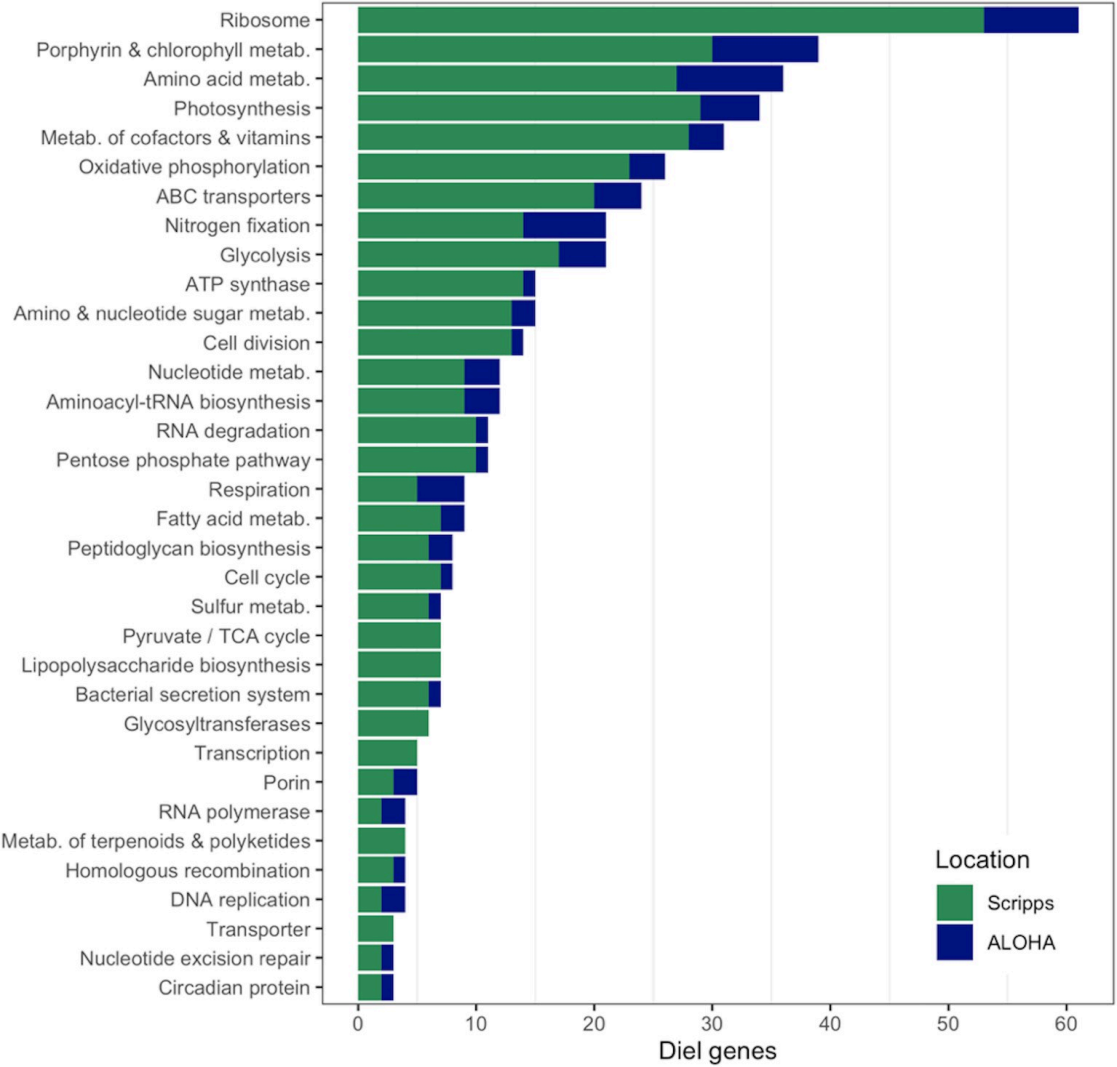
Abundance of UCYN-A1 and A2 diel genes at Stn. ALOHA (blue) and Scripps Pier (green). The *x* axis shows the number of diel genes detected from each pathway. For legibility only pathways with ≥3 diel genes are shown (450 of 459 total diel genes at either site).

Although many genes were detected at Stn. ALOHA, only ~10% (188 diel genes of 1939 detected genes) had significantly periodic ("diel") transcript levels, or 8%, 5.5%, and 8% of total known genes for UCYN-A1(96 diel genes of 1195), A2 (68 diel genes of 1244), and A3 (24 diel genes of 314), respectively (percentages based on the Diel column in S3 Table). In contrast, at Scripps Pier ~85% of detected genes from UCYN-A1 and A2 were diel (651 diel genes) (at least 27% of total known genes for each) [17] (Fig 4). The substantially lower percentages of diel genes in the present study persisted after controlling for different numbers of time points and detected genes in comparison to the coastal study (S1 File). Most of diel genes at both stations were involved in the same pathways such as ribosome biosynthesis, porphyrin and chlorophyll metabolism, amino acid metabolism, photosynthesis, and nitrogen fixation (Fig 4).

Several factors may explain why Stn. ALOHA had far fewer diel genes. The coastal samples were from the surface and likely experienced higher daily integrated light fluxes than Stn. ALOHA samples, which were from 45 m (14–19% of surface PAR; Letelier et al. 2004); total hours of light and dark were similar for both studies despite different seasons (S1 Table). At Stn. ALOHA, Vislova et al. (2019) observed clear decreases in percentages of diel genes from 25 m to 75 m, especially for haptophytes, when the mixed-layer depth was <75 m. Therefore, they attributed the overall proportions of diel genes to daily integrated light flux. Our 45 m samples were collected just below the mixed-layer depth (27.6±13.1 m, S2 Table) so our result is consistent with Vislova et al. (2019). Other factors may have contributed to the differences at Stn. ALOHA and Scripps Pier. Waters near Hawaii experience high levels of sunlight and warm temperatures year round while coastal California waters are colder and undergo marked seasonal transitions. Nutrient composition and organismal interactions may also be factors: Light may drive the release of organic matter by photosynthetic organisms at Stn. ALOHA, but land-derived organic matter is an additional factor at Scripps Pier.

We also checked whether the time of peak transcript levels changed for genes that were diel in both the NPSG and the coastal site ("cross-site diel genes"). Although nearly every diel gene at Scripps Pier was detected at Stn. ALOHA (649 of 651 diel genes), only 62 were cross-site diel (*n*_*A1*_ = 32, *n*_*A2*_ = 30; S5 Table). For both UCYN-A1 and A2, cross-site diel genes that peaked at sunrise (D12) at Scripps Pier also peaked at sunrise at Stn. ALOHA (S12 Fig). For UCYN-A1 these included genes for nitrogen fixation (*nifBESU*), the circadian oscillating protein 23 (COP23), and the metabolism of porphyrin and chlorophyll, which also always peaked at sunrise for UCYN-A2 (S12 Fig). Sunset (L12) peaks also usually persisted from Scripps Pier to Stn. ALOHA (for 5 of 6 UCYN-A1 genes (S12 Fig); UCYN-A2 had no cross-site diel genes that peaked at sunset; S5 Table). These included genes for the metabolism of amino acids (*glnA*), amino sugars/nucleotides (SQD1), and vitamin E. Interestingly, for both sublineages cross-site diel genes most often peaked in the afternoon (L9) at Scripps Pier (17 genes for UCYN-A1, 12 genes for UCYN-A2) but rarely peaked in the afternoon at Stn. ALOHA (one UCYN-A1 gene predicted to regulate disulfide bond formation) (S5 Table and Figs 3 and S10). For UCYN-A1, peaks often shifted from the afternoon (L9) at Scripps Pier to sunset at Stn. ALOHA, including genes for porphyrin and chlorophyll metabolism, nucleotide metabolism (*add*), and a ribosomal protein (RP-S4). A remarkable exception was the ATP synthase gene ATPF0C whose peak shifted from the afternoon at Scripps Pier to sunrise (D12) at Stn. ALOHA, synchronous with *nif* gene peaks. For UCYN-A2, 4 of the cross-site diel genes had peaks shift from the afternoon at Scripps Pier to sunrise at Stn. ALOHA, including genes for glycolysis (FBA) and the metabolism of cofactors and vitamins (*thiL*). Six of the remaining 8 genes had peaks shift to the morning (RNA degradation gene (*rnr*)) or noon (peptidoglycan biosynthesis [*vanY*], ferredoxin [*petB*]). Altogether these results suggest that for both UCYN-A1 and A2 the different conditions (e.g. light and nutrients) at Scripps Pier and Stn. ALOHA led to fewer genes with significantly periodic expression, and to shifts from mainly afternoon peaks at the coast to sunrise or sunset peaks at Stn. ALOHA.

These different conditions could affect UCYN-A directly or through the host. We suspect that the host played an important role because we observed inverse correlations for UCYN-A strains at the same location (Stn. ALOHA) to chlorophyll and oxygen (S7 Fig). Median correlations between chlorophyll and diel transcript levels from UCYN-A1, A2, and A3 were 0.56, -0.61, and 0.69, respectively (S7 Fig). Median correlations between oxygen and diel transcript levels were 0.60, -0.61, and 0.68 for UCYN-A1, A2, and A3, respectively. The CTD data reflect bulk measurements. We do not know the oxygen and chlorophyll levels experienced by UCYN-A in their respective photosynthetic, haptophyte hosts. However, the inverse correlations to photosynthesis variables, at the same location, suggest different interactions between UCYN-A sublineages and their respective hosts.

## Conclusions

This study provides the first comparison of metatranscriptomes from natural populations of UCYN-A in oligotrophic and coastal waters. Across environments and sublineages, UCYN-A had the same highly transcribed genes from indispensable pathways including photosynthesis, ATP synthases, N_2_ fixation, and respiration. Moreover, under conditions of lower light and nutrients UCYN-A maintained morning peaks for genes that underpin the exchange of fixed N_2_ with C from the host.

On the other hand, UCYN-A sensitivity to habitat was illustrated by the striking differences in transcription at coastal and oligotrophic sites. At the coastal site transcription patterns were similar across sublineages, but at Stn. ALOHA most genes had peaks shift, often to near sunrise or sunset, and sublineages took on distinct daily transcription schedules. The complex sublineage-specific responses at Stn. ALOHA, but not at the coastal site, point to host interactions as important drivers of UCYN-A transcriptomes. Further studies are needed to understand the regulation and detailed functions of genes that were differentially expressed between sublineages and sites. We hope that the patterns and responsive genes identified in the present work will be targets for studies of UCYN-A symbioses in culture, which has only recently become possible. Culture studies could show whether environmental changes impact host circadian rhythms, including which genes and metabolites might be affect the daily transcription cycle in UCYN-A.

## Supporting information

S1 Fig ERCC mRNA spike-in transcripts used to check for conservative gene detection. ERCC mRNA spike-ins are ordered by concentration (pre-amplification) along the x axis, which is roughly log-scale. The y axis indicates observed microarray intensities for ERCC probes, which are shown for all Stn. ALOHA samples (black points). The red line is the mean of the 95% quantiles for Agilent negative controls (mainly structural hairpins that should not hybridize). The green lines represent predicted intensities from a linear model based on ERCC concentrations (independent variable) and observed ERCC intensities. The upper green line is the predicted intensity for the least concentrated ERCC (00048_92 at far left), and the lower green line is the predicted intensity for 1 transcript (pre-amplification). The blue line shows the lowest intensity detected gene at Stn. ALOHA (Materials and Methods).
(TIF)

S2 Fig BLAST-based simulation of hybridization to probes. *In silico* hybridizations simulated Agilent SurePrint technology. Hybridization occurred if the BLAST high-scoring pair (HSP) aligned at >95%id and >95% of the 60 nt probe length.
(TIF)

S3 Fig Stn. ALOHA microarray design. All genes in the microarray are categorized by their pathway and UCYN-A sublineage. Note the different *x* axes for the upper and lower bar plots. A total of 1195 genes for UCYN-A1 and 1244 genes for UCYN-A2 were represented on the Stn. ALOHA and Scripps Pier arrays. The Stn. ALOHA array also included 314 known genes for UCYN-A3.
(TIF)

S4 Fig Venn diagram of orthologs for UCYN-A1, A2, and A3. All UCYN-A1, A2, and A3 genes with gene symbol and/or pathway annotation are represented. Hypothetical proteins are excluded. Orthologs are based on identical gene symbols between sublineages.
(TIF)

S5 Fig Stn. ALOHA samples were from below the mixed-layer. Pressure and density from CTD casts 11–20 are shown with colors indicating the corresponding time point from the microarray analysis. All 20 CTD casts were used to estimate the mixed-layer depth (mean = solid blue, s.d = dashed blue lines) based on a potential density offset of 0.03 kg/m^3^ relative to 10 dbar. Samples for metatranscriptomes were collected at 45 m (red line).
(TIF)

S6 Fig Times of day at which all detected genes from UCYN-A sublineages reached their lowest transcript levels at Stn. ALOHA and at Scripps Pier. For each habitat and sublineage, the histogram shows the time of lowest average transcript levels for each gene. The *y* axes show the percentage of detected genes from the sublineage and x axes the sampling time.
(TIF)

S7 Fig Correlations between transcript levels and environmental variables at Stn. ALOHA. For each UCYN-A sublineage at Stn. ALOHA, the violin plots show distributions of correlations between transcripts from diel genes (upper plots) and non-diel genes (lower) to environmental data. The metatranscriptomic and environmental data are from the same CTD casts (11–20) at 45 dbar. The median of each distribution is indicated by a solid black line. For diel genes, UCYN-A1 and A3 both have strong positive correlations to PAR and chlorophyll concentration, while UCYN-A2 has strong negative correlations.
(TIF)

S8 Fig Distributions of raw microarray probe intensities for each UCYN-A sublineage. Raw probe intensities from all Stn. ALOHA samples were pooled. Includes only probes that are <95% nid to any other probe (the vast majority) and therefore not expected to cross-hybridize among sublineages detected by Agilent SurePrint microarrays. The UCYN-A2 distribution suggests that UCYN-A2 transcripts were highly abundant, despite it being only ~0.7% of the UCYN-A population at Stn. ALOHA. *n* indicates the numbers of probes for each sublineage.
(TIF)

S9 Fig Correlations across sites of two UCYN-A sublineages. For each UCYN-A sublineage and pathway, the Pearson correlations across sites (NPSG and coastal) were calculated for the median transcript levels for the genes in the pathway (requiring ≥5 genes detected at both sites). The plot shows that 7 pathways (discs) had significantly correlated transcript levels (*p*<0.05) across sites for both UCYN-A1 and A2. Another 5 pathways (triangles) were significantly correlated across sites only for UCYN-A2.
(TIF)

S10 Fig At Stn. ALOHA, UCYN-A sublineages had different schedules for peak transcriptional activity. As in Fig 3, all detected genes were categorized by the time of their peak transcript level. At Stn. ALOHA, UCYN-A sublineages had a 3 h lag between the times when many of their genes peaked: D12 for UCYN-A3, L3 for UCYN-A2, and L6 for UCYN-A1. No sample was collected at D9 in the Scripps Pier study so that time point lacks an open circle.
(TIF)

S11 Fig NMDS of metatranscriptomes from Stn. ALOHA (left) and Scripps Pier (right) with fitted environmental data. For each site, the NMDS used transcript levels for 760 cross-site detected genes. The Stn. ALOHA NMDS included another 107 diel genes detected only at Stn. ALOHA and also shows fitted environmental data from CTD casts (Methods). Chlorophyll and oxygen concentrations were significantly correlated to transcript levels at Stn. ALOHA (p < 0.05, in red) and more highly correlated to genes for respiration and the metabolism of porphyrin and chlorophyll. Genes important to nitrogen fixation (nif genes, ATP synthases, oxidative phosphorylation and, hypothetically, Circadian Oscillating Protein 23) were anticorrelated with PAR and chlorophyll and had higher transcript levels near sunrise (2D12).
(TIF)

S12 Fig Diel genes at Stn. ALOHA mainly peaked at sunrise (D12) or sunset (L12), unlike at Scripps Pier. At Stn. ALOHA clustering assigned 154 of the 188 diel genes to the sunrise peak cluster (68 genes) or the sunset peak cluster (86 genes). For legibility only 53 diel genes are shown. Genes are colored by their diel cluster in the study at Scripps Pier [17]. Within each of the four plots genes with different colors appear, which indicates that genes with different diel schedules (clusters) at Scripps Pier changed to have the same diel schedule at Stn. ALOHA. At Scripps Pier 389 of the 651 diel genes were in cluster I (199 genes, L9 peak) or cluster II (190 genes, D12/L3 peak).
(TIF)

S1 Table Time points at which samples were collected for the Stn. ALOHA and Scripps Pier experiments. Sample labels indicate the number of hours in the light or dark period (Methods). Sunrise (L12) was at 6:01 at Scripps Pier and at ~6:18 at Stn. ALOHA. Replicates for each time point are described in Materials and Methods.
(XLSX)

S2 Table Environmental data for Stn. ALOHA and Scripps Pier. The first sheet has CTD data for the same casts that were used to collect samples for the Stn. ALOHA metatranscriptomes. The second sheet describes the daylight hours, mixed-layer depth, temperature, and salinity at Stn. ALOHA and Scripps Pier. For Stn. ALOHA, averages were calculated using CTD data at pressure 45 dbar from all casts during the cruise. For Scripps Pier, averages were calculated from morning measurements from July 2014. Data sources described in Materials and Methods.
(XLSX)

S3 Table Detected genes at Stn. ALOHA. For each of the 1939 detected genes at Stn. ALOHA, the normalized log_2_ transcript levels, annotation, and Fourier analysis results are provided.
(XLSX)

S4 Table Genes detected at both Stn. ALOHA and Scripps Pier. The 122 cross-site detected genes from UCYN-A1 and UCYN-A2 are listed with annotation.
(XLSX)

S5 Table Comparison of genes that had significant periodic ("diel") expression at both Stn. ALOHA and Scripps Pier. The first sheet categorizes the cross-site diel genes by the time of day at which they peaked at Stn. ALOHA versus at Scripps Pier. The second and third sheets describe the cross-site diel genes from UCYN-A1 and UCYN-A2, respectively.
(XLSX)

S6 Table Probe set sizes for the Stn. ALOHA and Scripps Pier microarray designs. Sheet 1 categorizes all genes by detection status and probe set size, for the Stn. ALOHA and Scripps Pier studies and their respective microarray designs. Sheet 2 categorizes genes in the Stn. ALOHA microarray design by pathway and probe set size. Sheet 3 does the same for the Scripps Pier design.
(XLSX)

S1 File (DOCX)
