# Supplementary figures and images for: Open ocean and coastal strains of the N2-fixing cyanobacterium UCYN-A have distinct transcriptomes
Source: PLoS One. 2023 May 2;18(5):e0272674. doi: 10.1371/journal.pone.0272674 (PMC10153697; doi:10.1371/journal.pone.0272674)

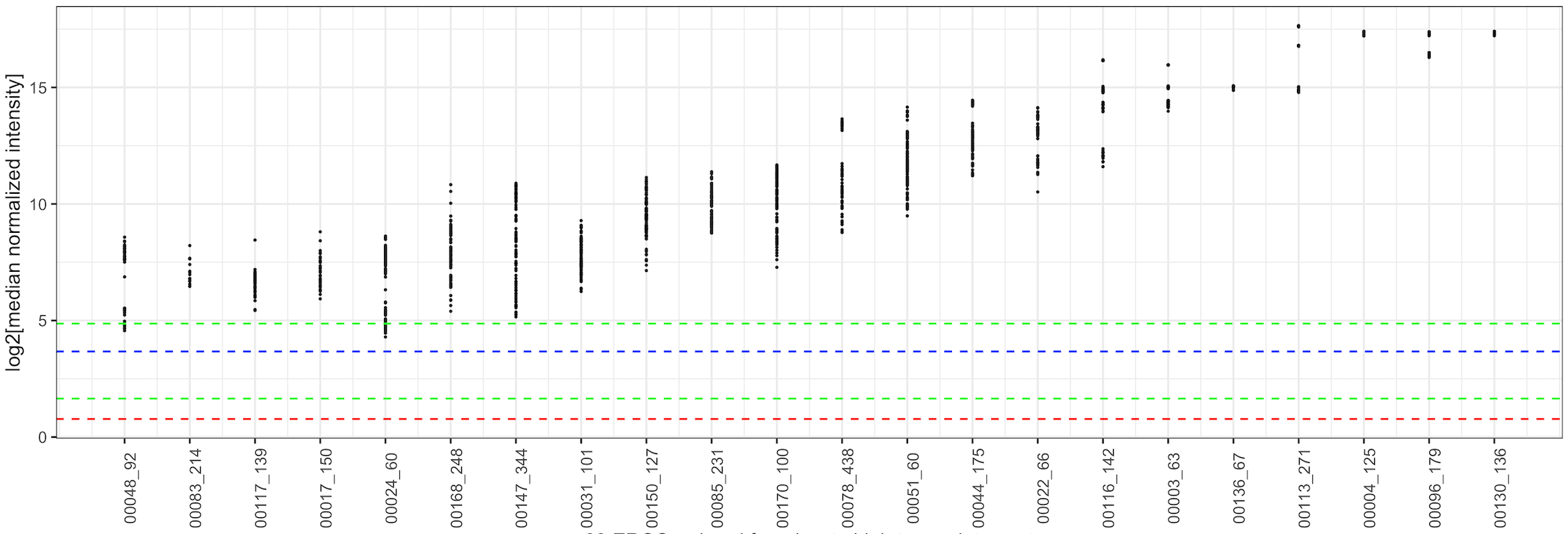

Supplement: S1 Fig — ERCC mRNA spike-ins are ordered by concentration (pre-amplification) along the x axis, which is roughly log-scale. The y axis indicates observed microarray intensities for ERCC probes, which are shown for all Stn. ALOHA samples (black points). The red line is the mean of the 95% quantiles for Agilent negative controls (mainly structural hairpins that should not hybridize). The green lines represent predicted intensities from a linear model based on ERCC concentrations (independent variable) and observed ERCC intensities. The upper green line is the predicted intensity for the least concentrated ERCC (00048_92 at far left), and the lower green line is the predicted intensity for 1 transcript (pre-amplification). The blue line shows the lowest intensity detected gene at Stn. ALOHA (Materials and Methods). (TIF) [file pone.0272674.s001.tif]

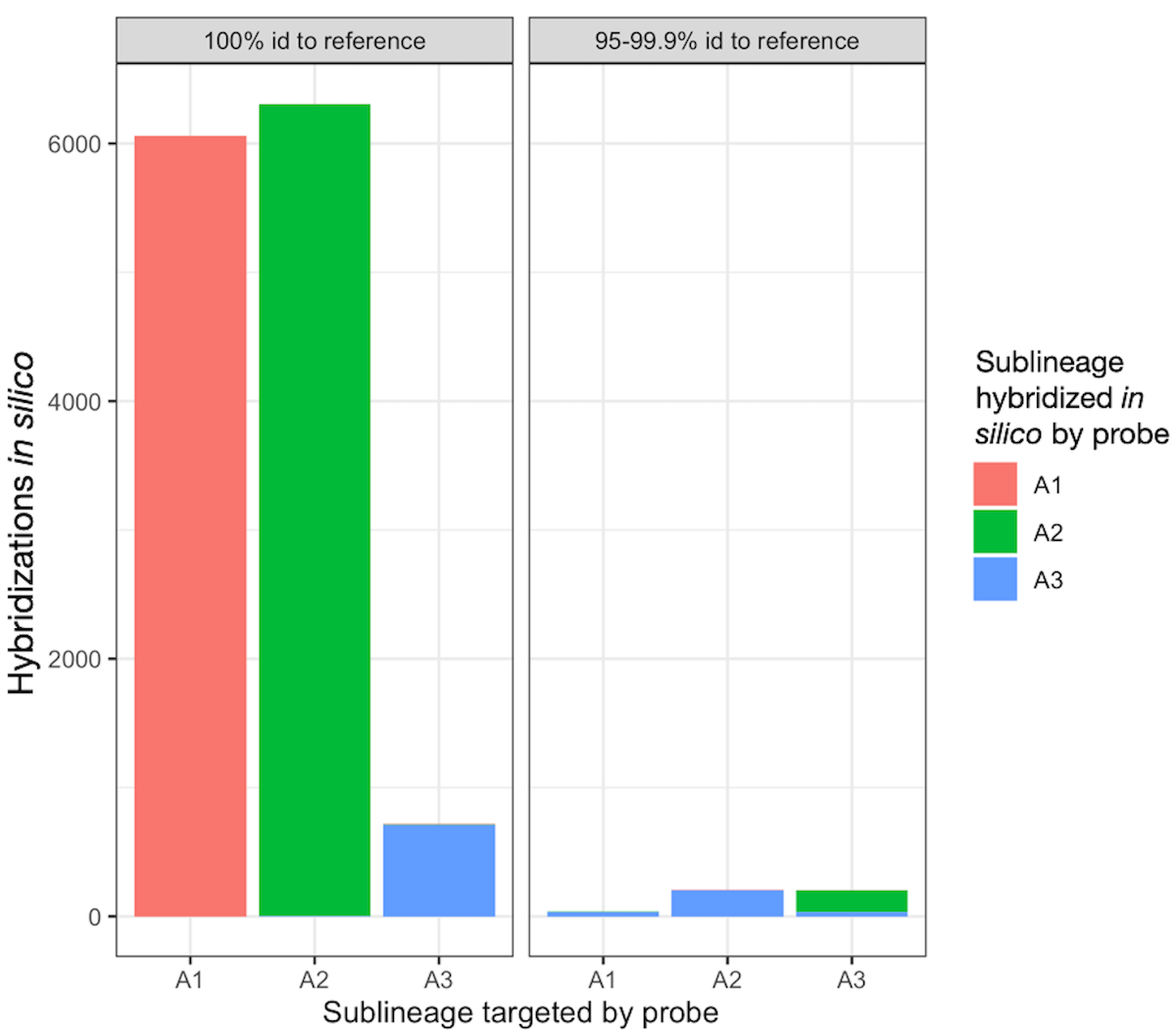

Supplement: S2 Fig — In silico hybridizations simulated Agilent SurePrint technology. Hybridization occurred if the BLAST high-scoring pair (HSP) aligned at >95%id and >95% of the 60 nt probe length. (TIF) [file pone.0272674.s002.tif]

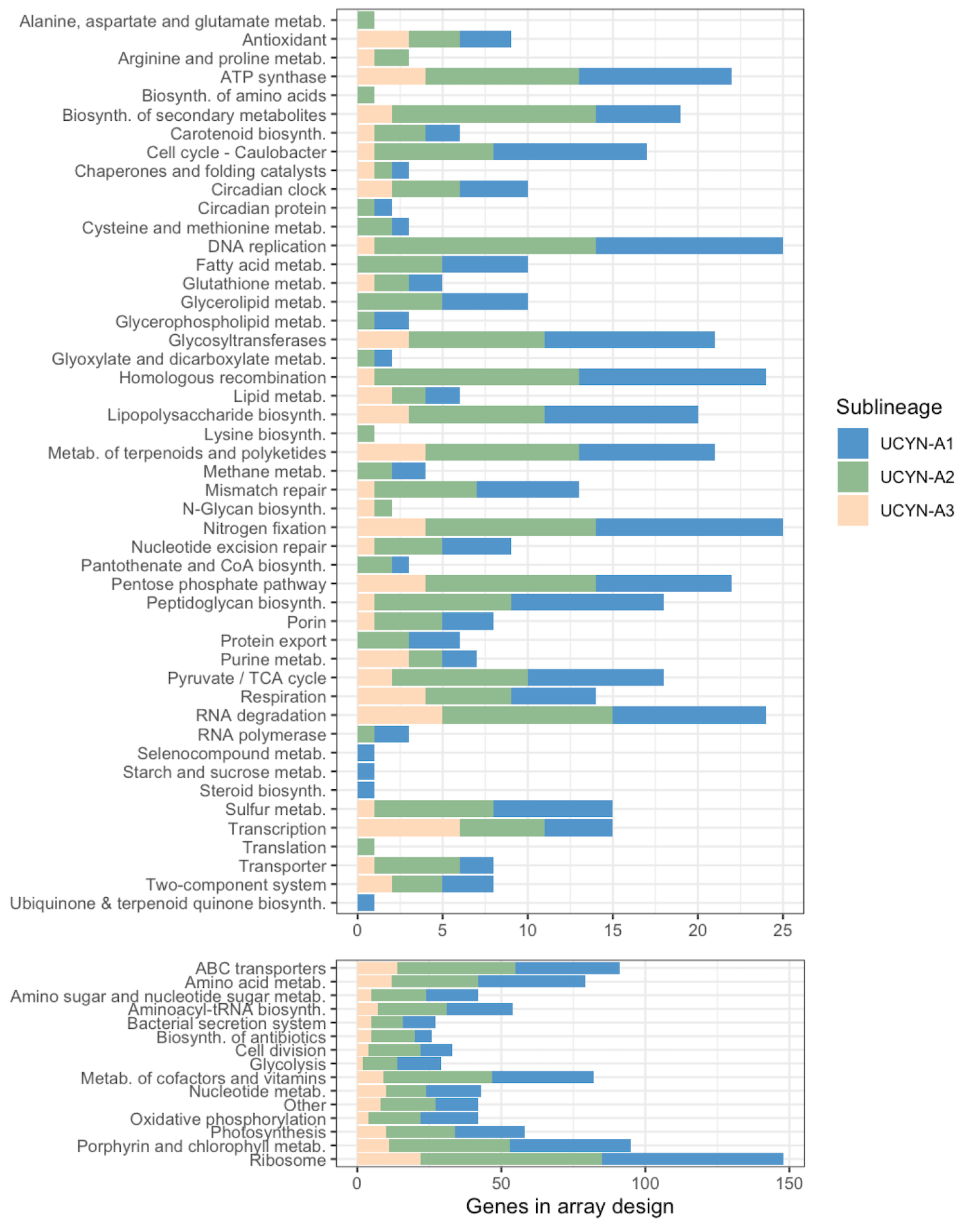

Supplement: S3 Fig — All genes in the microarray are categorized by their pathway and UCYN-A sublineage. Note the different x axes for the upper and lower bar plots. A total of 1195 genes for UCYN-A1 and 1244 genes for UCYN-A2 were represented on the Stn. ALOHA and Scripps Pier arrays. The Stn. ALOHA array also included 314 known genes for UCYN-A3. (TIF) [file pone.0272674.s003.tif]

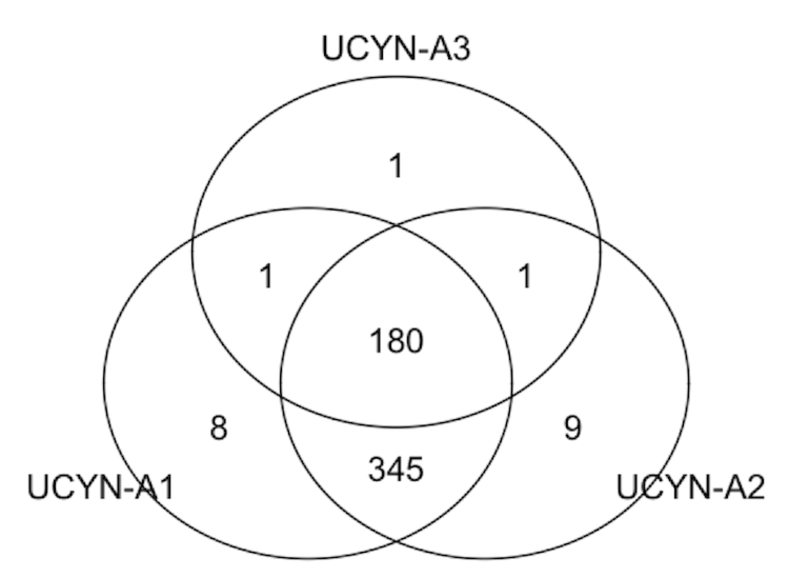

Supplement: S4 Fig — All UCYN-A1, A2, and A3 genes with gene symbol and/or pathway annotation are represented. Hypothetical proteins are excluded. Orthologs are based on identical gene symbols between sublineages. (TIF) [file pone.0272674.s004.tif]

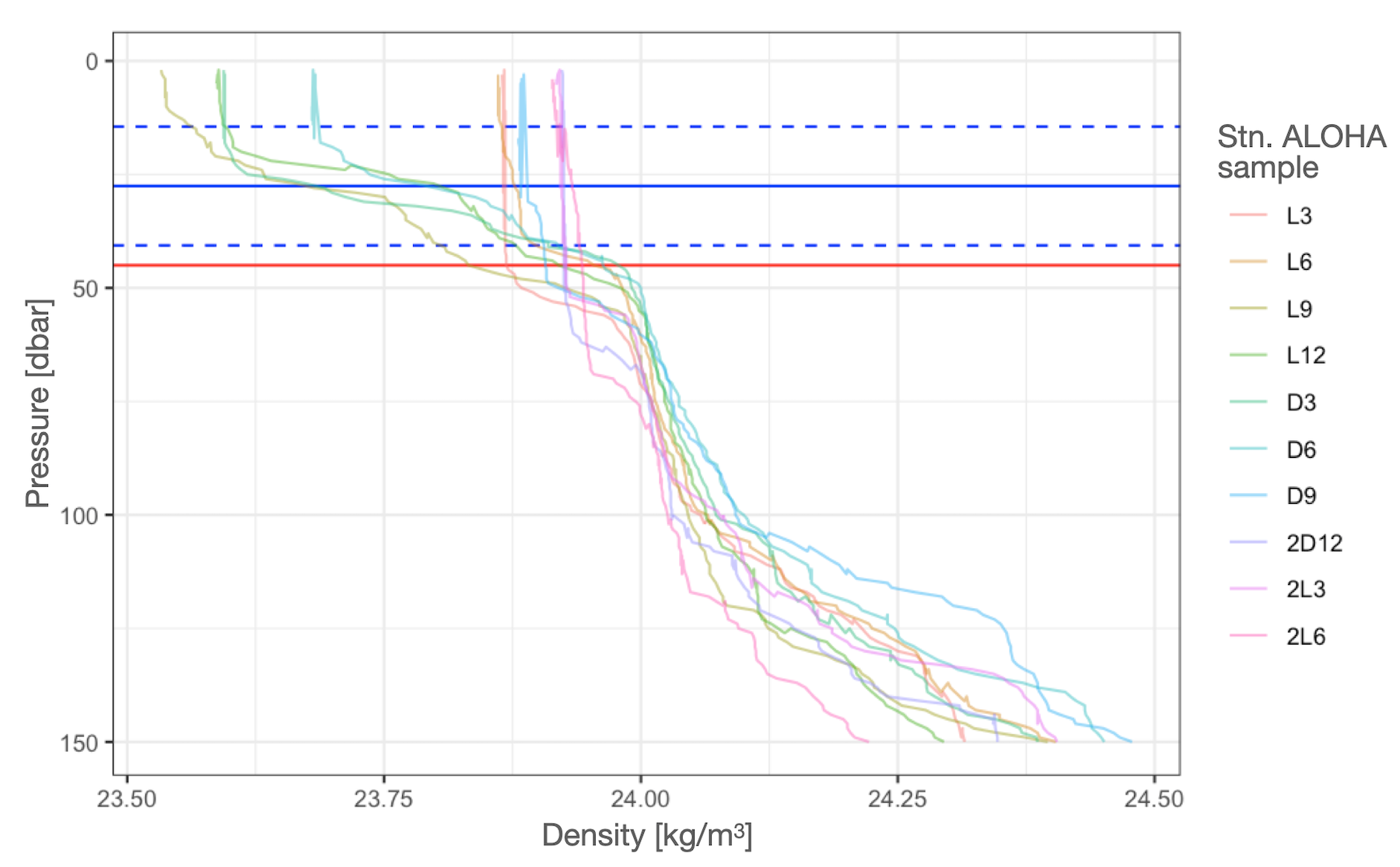

Supplement: S5 Fig — Pressure and density from CTD casts 11–20 are shown with colors indicating the corresponding time point from the microarray analysis. All 20 CTD casts were used to estimate the mixed-layer depth (mean = solid blue, s.d = dashed blue lines) based on a potential density offset of 0.03 kg/m3 relative to 10 dbar. Samples for metatranscriptomes were collected at 45 m (red line). (TIF) [file pone.0272674.s005.tif]

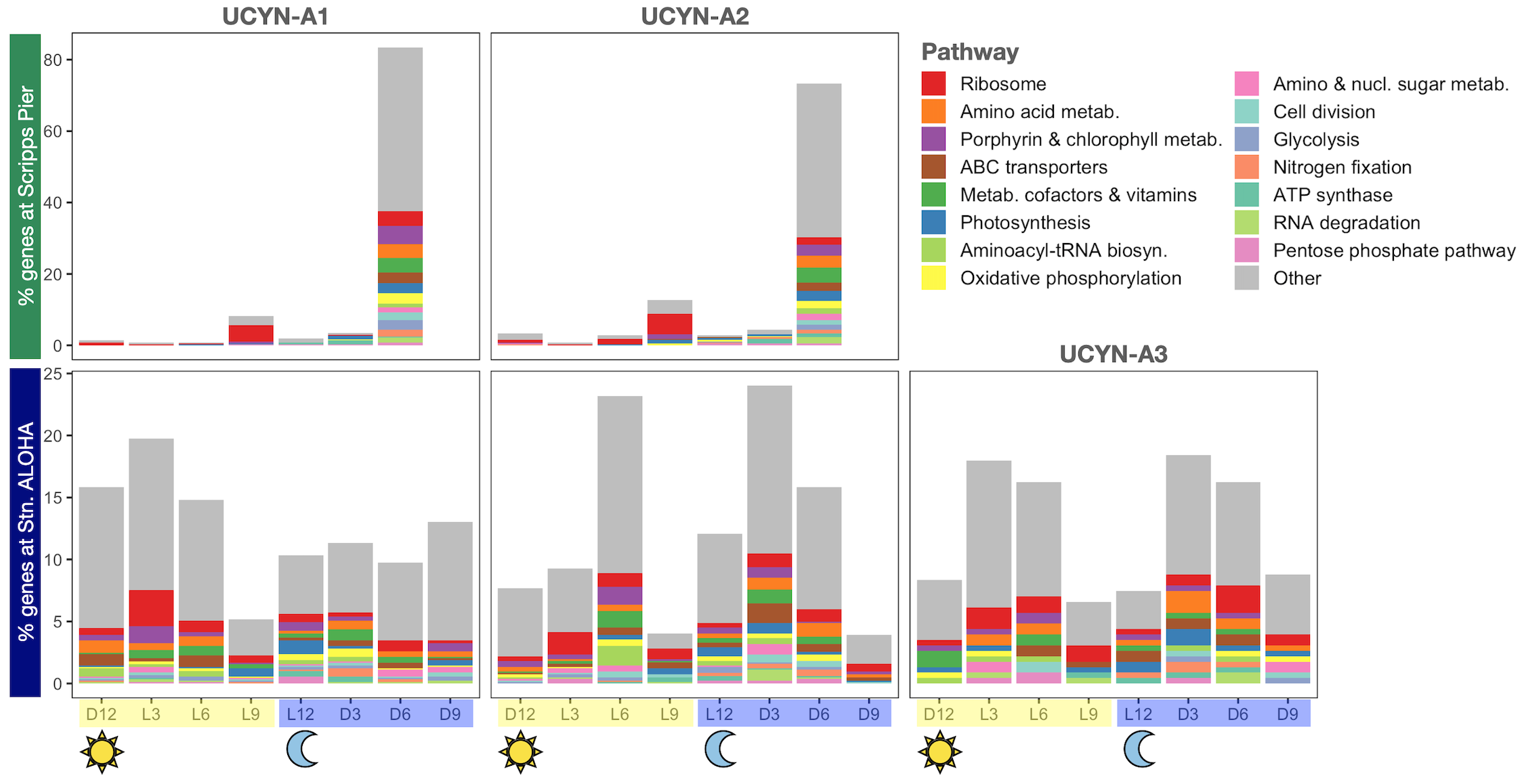

Supplement: S6 Fig — For each habitat and sublineage, the histogram shows the time of lowest average transcript levels for each gene. The y axes show the percentage of detected genes from the sublineage and x axes the sampling time. (TIF) [file pone.0272674.s006.tif]

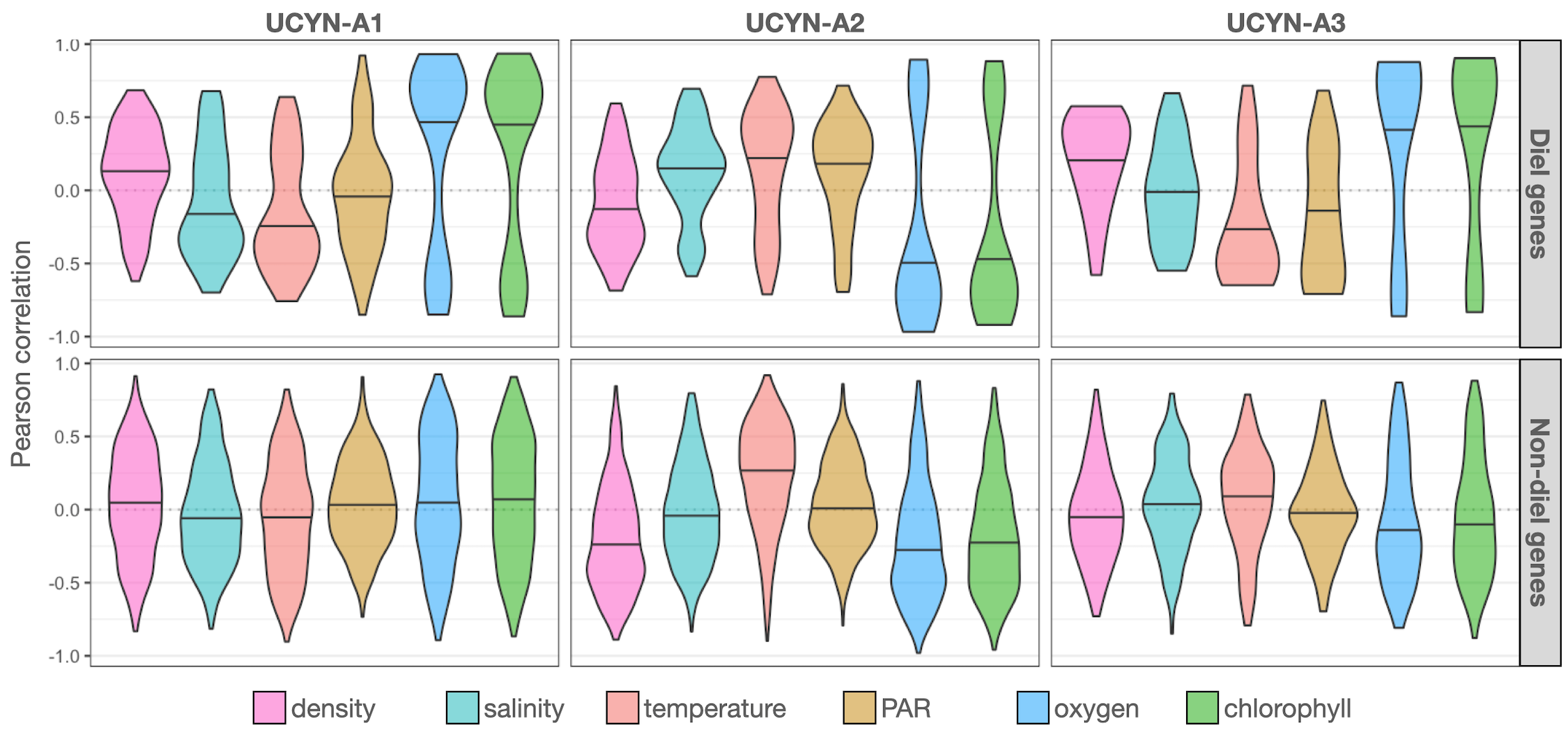

Supplement: S7 Fig — For each UCYN-A sublineage at Stn. ALOHA, the violin plots show distributions of correlations between transcripts from diel genes (upper plots) and non-diel genes (lower) to environmental data. The metatranscriptomic and environmental data are from the same CTD casts (11–20) at 45 dbar. The median of each distribution is indicated by a solid black line. For diel genes, UCYN-A1 and A3 both have strong positive correlations to PAR and chlorophyll concentration, while UCYN-A2 has strong negative correlations. (TIF) [file pone.0272674.s007.tif]

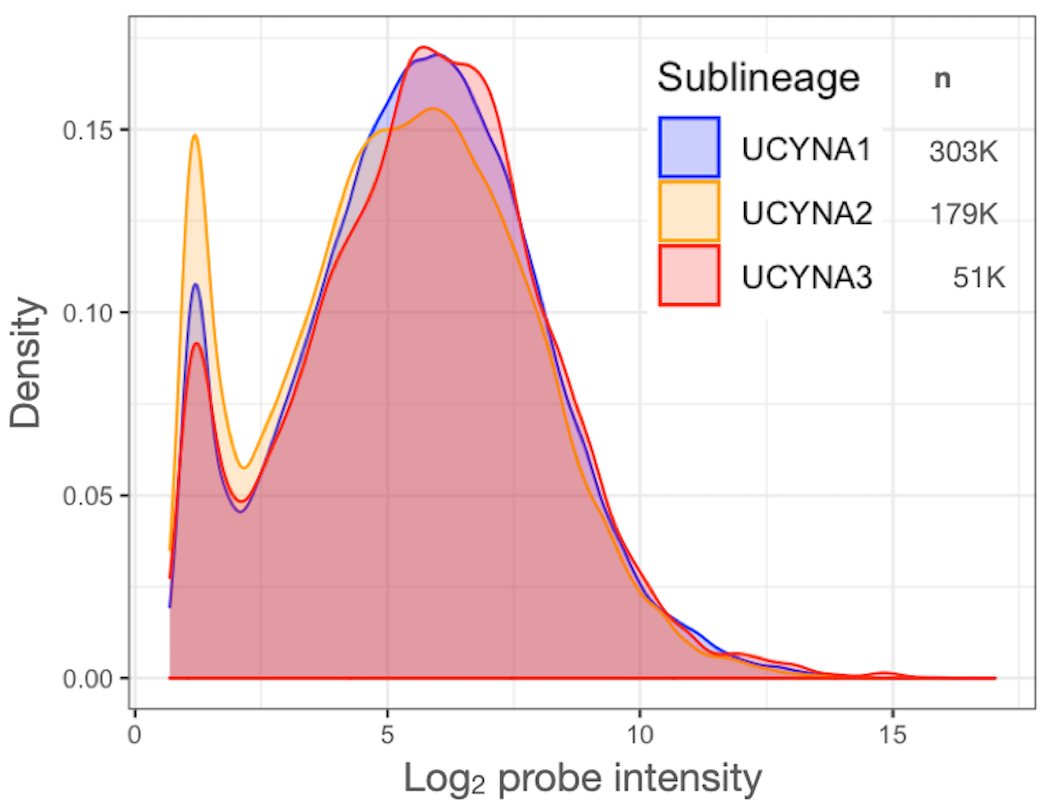

Supplement: S8 Fig — Raw probe intensities from all Stn. ALOHA samples were pooled. Includes only probes that are <95% nid to any other probe (the vast majority) and therefore not expected to cross-hybridize among sublineages detected by Agilent SurePrint microarrays. The UCYN-A2 distribution suggests that UCYN-A2 transcripts were highly abundant, despite it being only ~0.7% of the UCYN-A population at Stn. ALOHA. n indicates the numbers of probes for each sublineage. (TIF) [file pone.0272674.s008.tif]

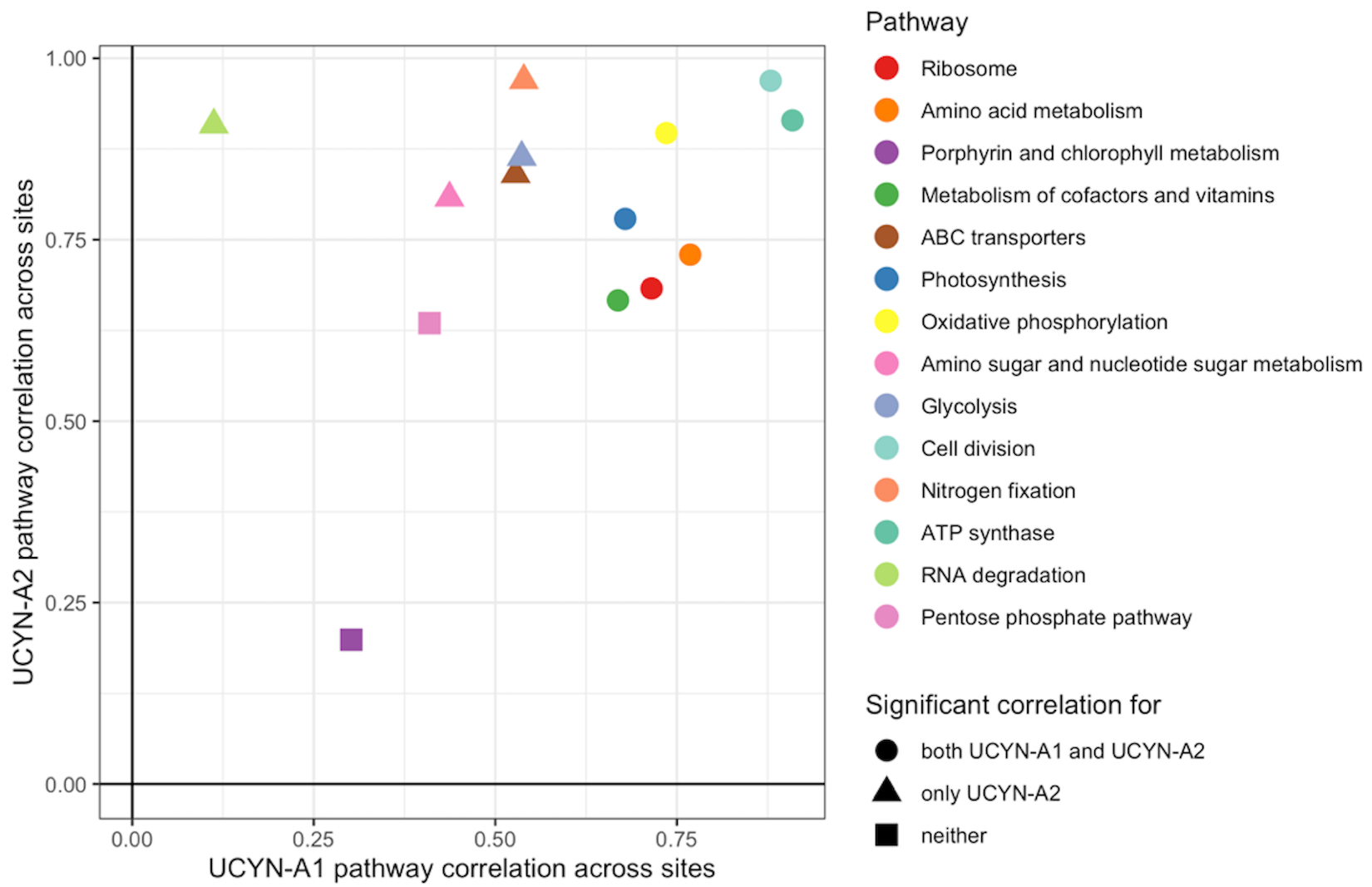

Supplement: S9 Fig — For each UCYN-A sublineage and pathway, the Pearson correlations across sites (NPSG and coastal) were calculated for the median transcript levels for the genes in the pathway (requiring ≥5 genes detected at both sites). The plot shows that 7 pathways (discs) had significantly correlated transcript levels (p<0.05) across sites for both UCYN-A1 and A2. Another 5 pathways (triangles) were significantly correlated across sites only for UCYN-A2. (TIF) [file pone.0272674.s009.tif]

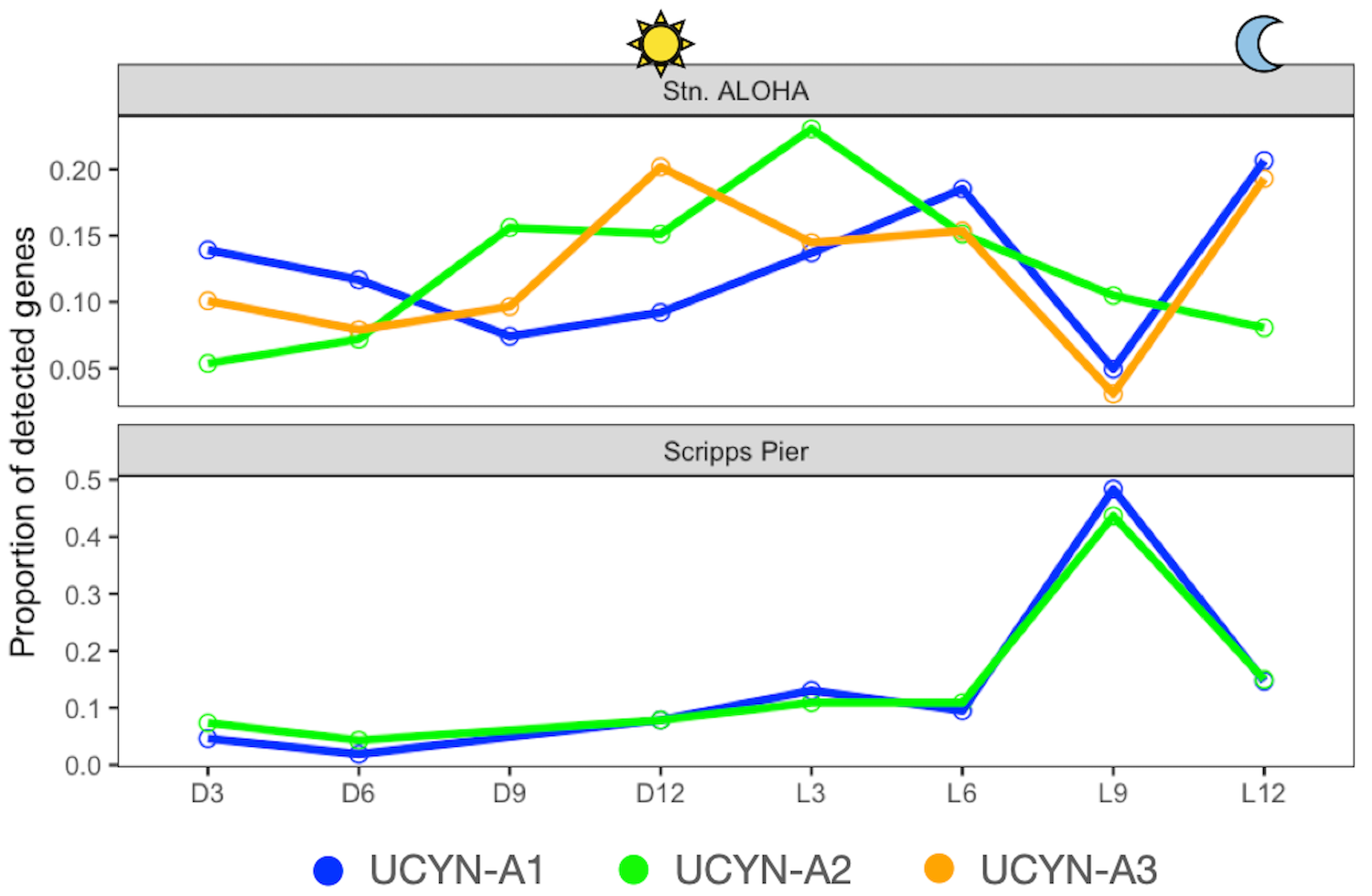

Supplement: S10 Fig — As in Fig 3, all detected genes were categorized by the time of their peak transcript level. At Stn. ALOHA, UCYN-A sublineages had a 3 h lag between the times when many of their genes peaked: D12 for UCYN-A3, L3 for UCYN-A2, and L6 for UCYN-A1. No sample was collected at D9 in the Scripps Pier study so that time point lacks an open circle. (TIF) [file pone.0272674.s010.tif]

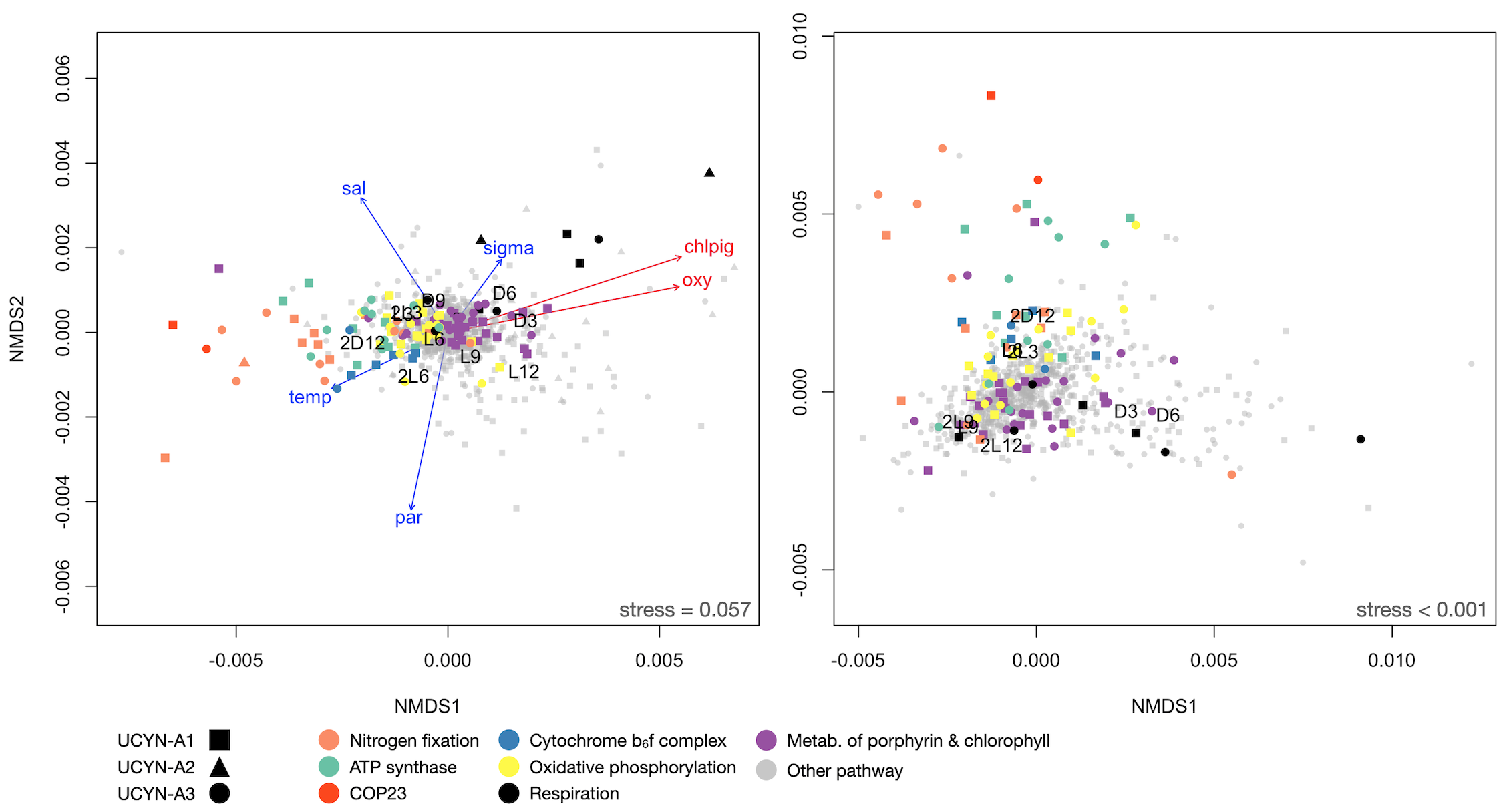

Supplement: S11 Fig — NMDS of metatranscriptomes from Stn. ALOHA (left) and Scripps Pier (right) with fitted environmental data. For each site, the NMDS used transcript levels for 760 cross-site detected genes. The Stn. ALOHA NMDS included another 107 diel genes detected only at Stn. ALOHA and also shows fitted environmental data from CTD casts (Methods). Chlorophyll and oxygen concentrations were significantly correlated to transcript levels at Stn. ALOHA (p < 0.05, in red) and more highly correlated to genes for respiration and the metabolism of porphyrin and chlorophyll. Genes important to nitrogen fixation (nif genes, ATP synthases, oxidative phosphorylation and, hypothetically, Circadian Oscillating Protein 23) were anticorrelated with PAR and chlorophyll and had higher transcript levels near sunrise (2D12). (TIF) [file pone.0272674.s011.tif]

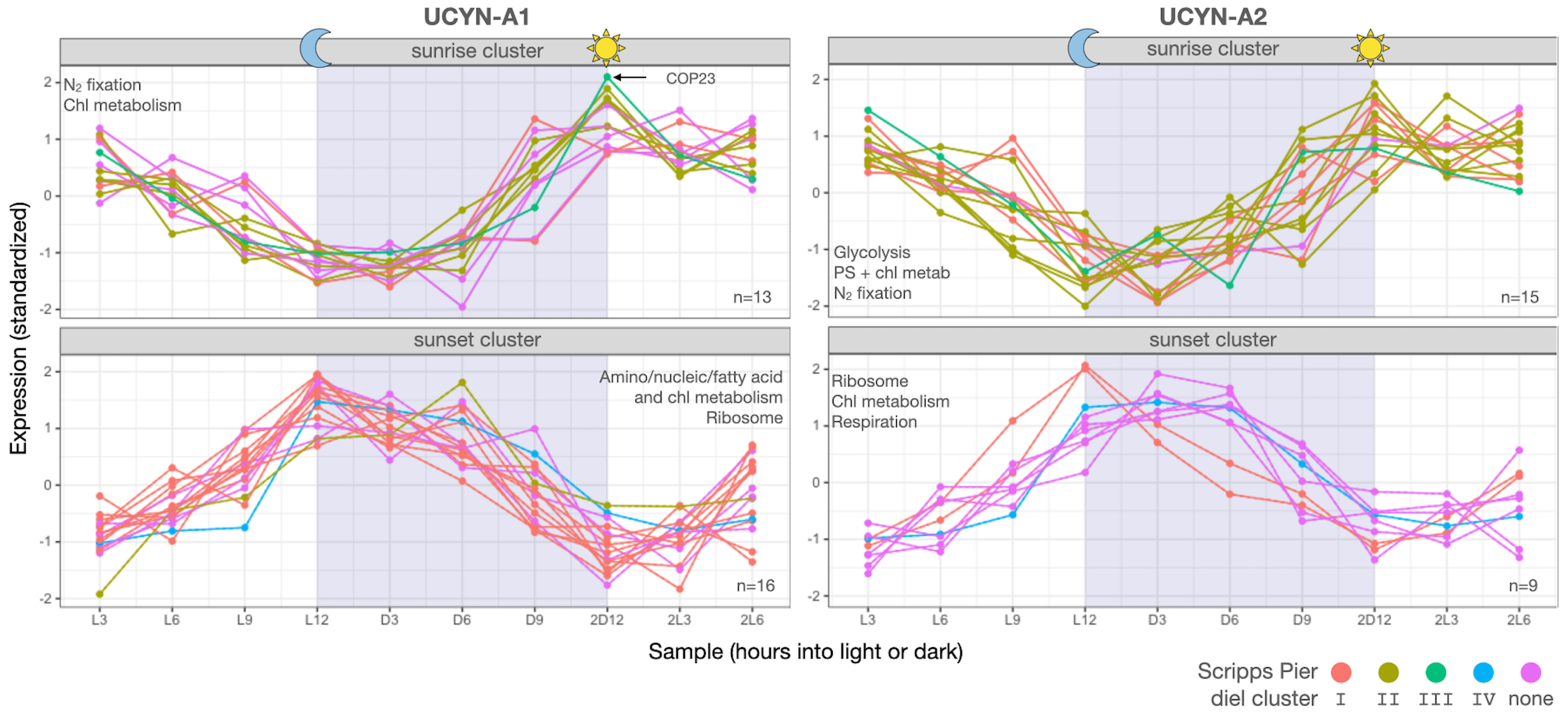

Supplement: S12 Fig — At Stn. ALOHA clustering assigned 154 of the 188 diel genes to the sunrise peak cluster (68 genes) or the sunset peak cluster (86 genes). For legibility only 53 diel genes are shown. Genes are colored by their diel cluster in the study at Scripps Pier [17]. Within each of the four plots genes with different colors appear, which indicates that genes with different diel schedules (clusters) at Scripps Pier changed to have the same diel schedule at Stn. ALOHA. At Scripps Pier 389 of the 651 diel genes were in cluster I (199 genes, L9 peak) or cluster II (190 genes, D12/L3 peak). (TIF) [file pone.0272674.s012.tif]
