# Supplementary material for: Open ocean and coastal strains of the N2-fixing cyanobacterium UCYN-A have distinct transcriptomes
Source: PLoS One. 2023 May 2;18(5):e0272674. doi: 10.1371/journal.pone.0272674 (PMC10153697; doi:10.1371/journal.pone.0272674)
Supplement: S1 File — (DOCX) [file pone.0272674.s019.docx]

**Supplementary Information**

**Impacts of different probe set sizes to detected genes and pathways**

We checked whether the sizes of probe sets (the number of probe sequences representing a gene on the microarray) impacted gene detection. Most (91%) of the 2753 genes in the Stn. ALOHA microarray design and 91% of the 2431 genes in Scripps Pier microarray design had probe sets with ≥3 probes (S6 Table). Pathways represented by genes with <3 probes were well-represented by genes with ≥3 probes. Thus, the probe set size did not likely impact gene detection or pathway analyses (S6 Table).

**Fewer diel genes were detected at Stn. ALOHA compared to Scripps Pier**

We checked whether the lower number of diel genes at Stn. ALOHA (188 total) compared to Scripps Pier (651 total) was due to differences between the two studies in the number of time points (10 versus 8, respectively) or detected genes (1939 versus 762, respectively). To control for these differences, we reran the Stn. ALOHA Fourier score analysis (Materials and Methods) using the 8 shared time points and 760 genes detected in both studies. The normalized analysis also identified far fewer diel genes at Stn. ALOHA (106 genes versus 651 in the coastal study).
